# A Simple Extension to the CMASA Method for the Prediction of Catalytic Residues in the Presence of Single Point Mutations

**DOI:** 10.1371/journal.pone.0108513

**Published:** 2014-09-30

**Authors:** David I. Flores, Rogerio R. Sotelo-Mundo, Carlos A. Brizuela

**Affiliations:** 1 Departamento de Ciencias de la Computación, Centro de Investigación Científica y de Educación Superior de Ensenada, Ensenada, Baja California, México; 2 Centro de Investigación en Alimentación y Desarrollo, A.C., Hermosillo, Sonora, México; University of Rome Tor Vergata, Italy

## Abstract

The automatic identification of catalytic residues still remains an important challenge in structural bioinformatics. Sequence-based methods are good alternatives when the query shares a high percentage of identity with a well-annotated enzyme. However, when the homology is not apparent, which occurs with many structures from the structural genome initiative, structural information should be exploited. A local structural comparison is preferred to a global structural comparison when predicting functional residues. CMASA is a recently proposed method for predicting catalytic residues based on a local structure comparison. The method achieves high accuracy and a high value for the Matthews correlation coefficient. However, point substitutions or a lack of relevant data strongly affect the performance of the method. In the present study, we propose a simple extension to the CMASA method to overcome this difficulty. Extensive computational experiments are shown as proof of concept instances, as well as for a few real cases. The results show that the extension performs well when the catalytic site contains mutated residues or when some residues are missing. The proposed modification could correctly predict the catalytic residues of a mutant thymidylate synthase, 1EVF. It also successfully predicted the catalytic residues for 3HRC despite the lack of information for a relevant side chain atom in the PDB file.

## Introduction

The automatic annotation of protein functions is a challenging problem in structural bioinformatics. The ambiguous definition of protein function itself makes the problem even more complicated [1], [2]. Function considers many aspects: biochemical, physiological, cellular, and medical. Given this complexity, it is practically impossible to develop predictive models based on sequence, structure, or any other descriptive characteristics of the protein for a general case. Therefore, predictors that focus on specific aspects related to function seems more promising.

Enzymatic functions are utilized in almost all biological processes; these functions are usually related to a few catalytic residues. The automatic identification of these relevant residues has been approached from many contexts. When the sequence identity between the query protein and some known enzyme is high, sequence-based methods, such as BLAST [3], PSI-BLAST [4], or PROSITE [5], may work. However, when there is not close homology, structure-based methods should be used.

The Structural Genomics Initiative [6], which contributed more than a thousand new structures to the PDB, has accelerated the development of structure-based prediction methods for function in general [7] and for catalytic residues in particular [8]–[11]. These methods are based on the assumption that enzyme functions are determined by a group of a few residues in the active site. Thus, two proteins with similar functions should have similar local patterns in the corresponding active sites, regardless of the sequence or the global structure. These methods take a predefined pattern associated with a particular function and search for this pattern in the structure of the query protein. Our proposal falls within this group of methods.

Most of these local structure-based methods use a database of patterns that are assumed to be responsible for a specific function. The methods search for these patterns and transfer the function from the pattern that best matches the query protein. The patterns in the database can be found using existing methods designed for this search [12]–[15] or using experimentally obtained information about the active sites, such as the information from the Catalytic Sites Atlas (CSA) [16].

One of the first methods in this category was ASSAM [17]. In this approach, a graph is built for the query protein structure, and another is built for the pattern. Next, Ullman’s subgraph isomorphism algorithm [18] is used to determine whether the pattern sub-graph is contained within the graph of the query protein. To generate the graphs, each residue in the pattern and in the protein is represented by two pseudo-atoms (S and E), which indicate the start and end of the functional group of the side chain. These atoms become the nodes of a 3D graph, and each node is labeled with the amino acid type and the type of pseudo-center (S or E). That is, the method considers the position, orientation, and type of residue. However, a newer version [19] also accounts for the solvent accessibility, the type of secondary structure of the residue, and the disulfide bonds, among other improvements. The method was easily able to find patterns of catalytic triads for some serine proteases and other common patterns in enzymes.

TESS [20] uses geometric hashing to search for the common structural patterns responsible for a function in all enzymes in the PDB. Some of the identified patterns were catalytic triads and active sites in ribonucleases and lysozymes. The method is divided into two phases: preprocessing and template search. In the first stage, all PDB structures are preprocessed. To achieve this aim, each amino acid is represented by three atoms, and a hash key is created for each pair of atoms that are within a threshold distance of each other. In each slot of the table, the relative positions of close atoms are stored. During the template search stage, all patterns are checked against all entries in the table, and those entries with the largest number of coincidences indicate the presence of the pattern in the protein.

As an improvement to TESS, a method known as JESS [21] was proposed. This procedure allows for the inclusion of arbitrary geometric and chemical constraints in the definition of the obtained templates from the database. This addition results in more flexible models than those generated with TESS.

THEMATICS [22] is a method that finds catalytic residues when a protein has a known structure but does not show any sequence or structure similarity to other proteins. It does not require homology or any other extra information besides the structure of the query protein. It is based on the physicochemical properties of the residues and its neighbors.

PINTS [23] performs database searches for user-specified patterns and provides a measure of the statistical significance of the outcomes. Using PINTS, previously computed patterns can also be compared to databases of complete structures, databases of entire structures, and databases of particular residues that are likely to be functionally important, as is the case for catalytic residues.

eF-Site [24] is a method that considers the electrostatic potential, the geometry and the hydropathy on the protein surface as criteria to measure the similarity between regions of local structures. This method maintains a database with information regarding the molecular surface of active sites with known function. The method creates graphs for the structure and for the pattern, where the vertices are points on the surface; the vertices are labeled with the electrostatic potential and the hydrophobicity, among other characteristics. From the two graphs, a third graph is generated where the nodes are all pairs of nodes in the structure pattern and in the query with similar labels. These nodes are connected through an edge if the corresponding distances are similar in both graphs. In the last step, the Bron-Kerbosch algorithm is used to search for a maximum clique [25]. The clique represents the largest common structural pattern between the protein and the local structure. The method was able to find local similarities beyond the global structure.

SiteEngine [26] is also designed to find regions on the protein surface similar to a specific binding site of a given protein. The representation is the one proposed in CavBase [27]. In this method, each residue is represented by generic pseudo-centers, which codify the physicochemical properties that are important for molecular interactions.

Query3d [28] is a method to find local structural motifs that are commonly found in proteins. Query3d is also a database management system. It can search for similar regions between the following: two protein structures, a structure and all structures in the PDB, and subsets of arbitrary amino acids within a structure.

An accurate and fast method for the prediction of catalytic residues is the one known as CMASA [29]. CMASA implements an algorithm that compares a database of catalytic residues and their 3D structure with the query protein. Each amino acid is represented using information about the alpha-carbon atom (*Cα*) and the side chain atom that is furthest from the *Cα*, denoted as *fa*. These atoms are labeled with the amino acid type. The method obtains its templates from the CSA and the PDB, and the pattern is modeled as two distance matrices: one for all-pairs distances of the *Cα* of all catalytic residues and the other for all pairs of the *fa* atoms. Another recent approach that follows similar ideas is CatSId [11]; both approaches use distance matrices to represent the pattern. However, in CMASA two matrices are used, whereas CarSId uses only the matrix of distances related to the *Cα*. The main difference, however, is that CMASA is a similarity-based approach and CatSId is a machine learning-based approach.

The work we propose here is an extension to CMASA to efficiently account for single point substitutions or missing data in the PDB entry. Although CMASA was designed to address single point mutations by using a substitution matrix, we show that the implementation does not work in many cases. The proposed idea is simple and consists of extending the template library to consider all *n−1* combinations of the *n* catalytic residues of each active site. In this manner, the extension is able to recover the non-mutated residues of the catalytic site without substantially degrading its performance for non-mutated cases.

## Materials and Methods

### The test sets

Before introducing the test sets, the concepts of template and master template must be introduced. A template is defined as the set of 3D coordinates of each alpha carbon (*Cα*) and the furthest atom (*fa*) in the side chain of every catalytic residue of a given enzyme. For a given family, a master template is defined as the template that minimizes the following expression [29]:
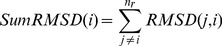
(1)Here, *RMSD(i,j)* is the RMSD between the *i*th and *j*th templates in the *r*th family, and *n_r_* is the number of templates in the family.

Two test sets (A and B) are proposed. Each of these test sets contains a positive group of proteins (i.e., proteins whose catalytic residues are annotated in the CSA) and a negative group (proteins that have no catalytic function and therefore do not have inputs in the CSA). For both test sets, the negative group is the same. This negative set is made of 10575 structures from the nrPDB, and it was provided by the authors of CMASA. The test sets A and B differ in the composition of their positive groups. To make the document self-contained, we describe how the authors of CMASA [29] generated their test set. This test set is equivalent to our test set A and was generated as follows:

1. All proteins in the PDB were considered; then, using blastclust (from BLAST), clusters that grouped proteins with a sequence identity of 90% or more were generated. From each of these clusters, the protein with the highest resolution was selected as the representative of the cluster. The resulting set of proteins is termed nrPDB.
2. The positive group is obtained from the intersection of nrPDB and the proteins annotated in the CSA. This group is composed of 868 proteins, and these proteins were grouped according to their EC number.
3. Proteins in the nrPDB with no entries in the CSA compose the negative group with 10575 structures.

Next, we describe the positive groups for test sets A and B.

#### Positive group in test set A

This positive group is taken from the supplementary material of CMASA [29], which lists 868 proteins that have at least three catalytic residues each; these proteins are distributed in 164 families. Starting from this set, we recovered 744 proteins in 163 families. The 124 excluded proteins show one of the following characteristics:

- Differences in the number of catalytic residues among members of the same family and their master template. For instance, in the supplementary material of CMASA [29], the master template 1DXL (lipoamide dehydrogenase) has four catalytic residues C45-C50-H449-E454; however, in the database for the software [29], the 3D coordinates for five residues C45-C50-T215-H449-E454 are given, i.e., residue T215 is added. The following enzymes belong to the family of 1DXL: 1EBD, 1JEH, 1LPF, 1LVL, 1ZMC, 2A8X, and 3LAD; however, all of them have four catalytic residues in the CMASA database but five residues in the master template. Due to this difference in the number of atoms between the master template and the members of the family, CMASA associates each of these proteins with a different master template (1GER) instead of matching them to the template of 1DXL.
- Differences in amino acid types: The standard configuration of CMASA performs an incorrect prediction when the master template and the query protein belong to the same family but have at least one different residue. For instance, the catalytic residues of the master template 1E7P (fumarate reductase) are H257-E294-R301-H369-R404. However, protein 1KF6, which belongs to the same family, has the following residues H232-G269-R287-H355-R390. Because of the difference between the residues E294 and G269, CMASA is not able to predict the catalytic site. Similar outcome results occur for 1NEK, a member of the same family.

The list showing each excluded protein and the reason for its exclusion is given in Table S1, it also lists the 163 resulting families with a total of 744 proteins.

#### Positive group in test set B

This group is composed of a set of mutated proteins from the positive group in test set A (744 proteins). First, we filtered all proteins with three catalytic residues; this process generated a group of 480 enzymes belonging to 108 families (from 163). Second, random point mutations were applied to each catalytic site. The chosen residues were substituted by alanine (alanine scanning). The computational mutagenesis of residues to alanine is among the fastest methods to validate hypotheses about protein function and test methods [30]. The set of mutated proteins along with the residue that is mutated in each protein are listed in Table S2.

### Similarity measures and criteria for performance evaluation

- CMAD (Contact Matrix Average Deviation) [29]: This is a similarity measure for the distances of the *n_ti_* atoms belonging to a template *t_i_* and those belonging to the local structure *lq*.
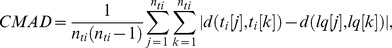
(2)where *d(ti[j], ti[k])* is the distance between atoms *j* and *k* from template *ti*, and *d(lq[j], lq[k])* is the distance between atoms *j* and *k* from the local structure *lq*. For the algorithm to identify the local structure of the query as a match for a given template, the CMAD of their corresponding *Cα* and their *fa* atoms should be smaller than or equal to a given threshold that can be tuned by the user.
- Statistical significance: The p-value computation used in CMASA was proposed by Stark et al. [31]. The idea is to assess the statistical significance of finding a structural match between the residues in the template and the corresponding residues in the query. These residues in the template as well as those in the query, are not necessarily adjacent in the protein sequence. The significance p-value (see equation 3) is assessed using an extreme value distribution (EVD), similar to the assessment in database searches. The p-value is calculated from an expectation function (EF(*R_M_*)) that estimates the number of matches in the whole query structure that have a score equal to or better than *R_M_*. In the case of CMASA, the score is given by the RMSD that is computed between the residues in the template and the residues in the query structure. In *EF* (see equation 4), *θ* is the product of the percentage abundance of all residues. The constants in *EF* were derived by Stark et al. [31] from an analysis of the searches for a typical structure, as named by the authors, against a background database composed of 723 folds from a non-redundant structure database. The p-value gives the probability of randomly obtaining an RMSD between a local structure and the template that is less than or equal to the RMSD between the template and the local structure predicted by the method. A statistical significance threshold (p-value = 1.0 E-4) and the exact match of all catalytic residues in *t_i_* were the criteria used to assess the results obtained by our proposed extension.

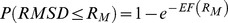
(3)

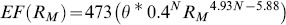
(4)

- True positive (*TP*): a query protein of the positive type that exactly matches a master template (*t_i_*) and has a p-value below the threshold.
- False negative (*FN*): For a query protein of the positive type, the prediction does not find a match to the same catalytic residues of the query protein or the match site has a p-value larger than the threshold.
- False positive (*FP*): For a query protein of the negative type, the prediction indicates a match with any master template, and the p-value for the match site is below the threshold.
- True negative (*TN*): For a query protein of the negative type, the prediction does not find a match to any master template with a p-value below the threshold.

Based on these definitions we use the following performance criteria:

- Sensitivity [32]:
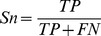
(5)
- Accuracy:
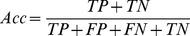
(6)
- Precision:
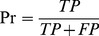
(7)
- MCC: the Matthews correlation coefficient [33]:
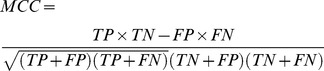
(8)

### Extending the CMASA method

**Figure 1:**
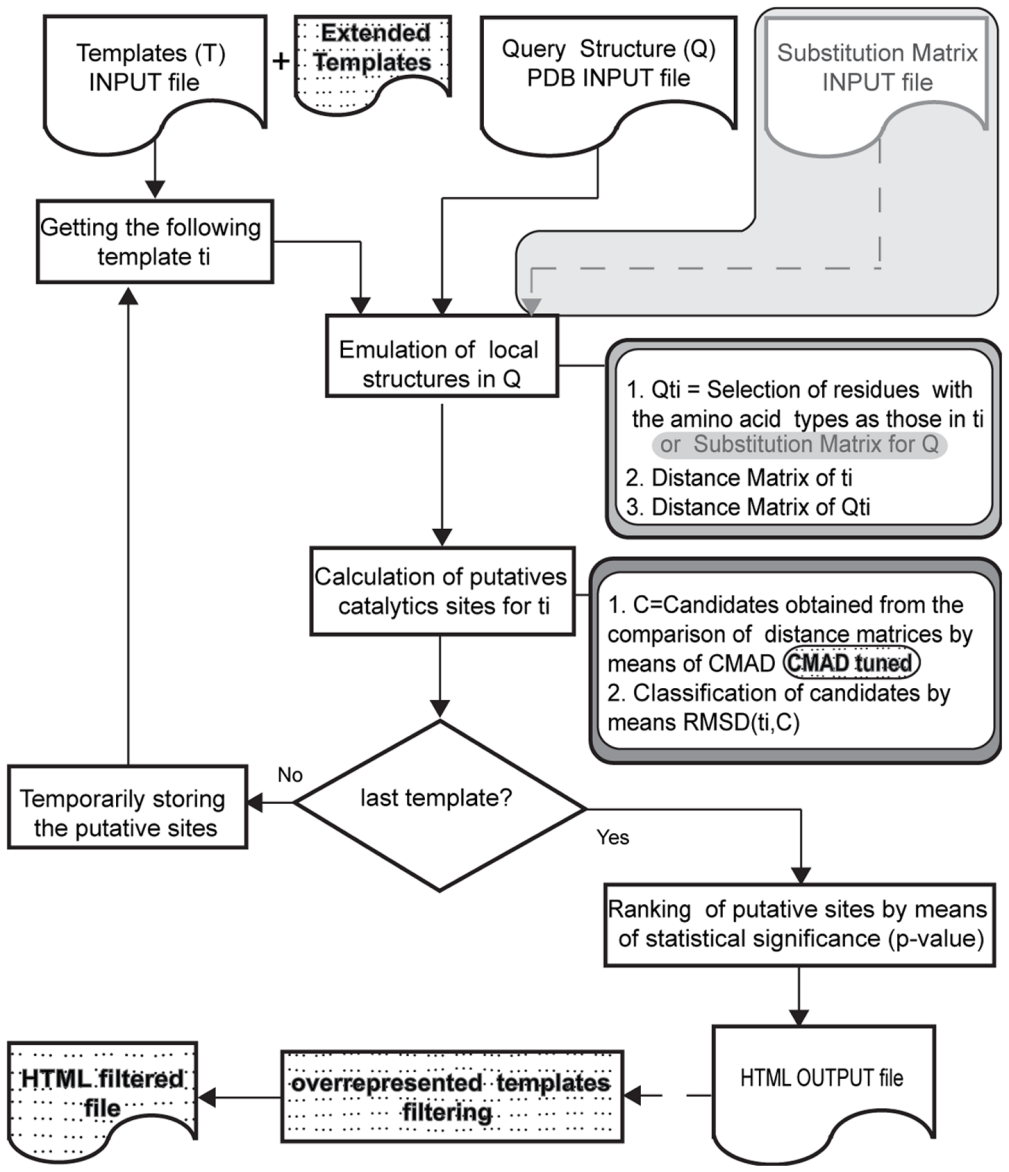
Flowchart of CMASA and the proposed extension. The diagrams with a dashed background indicate added characteristics, the grey shadowed regions correspond to the process of introducing the SM, and the dashed arrows indicate optional flow.

The general flowchart for CMASA and the proposed extension are shown in Figure 1. To better understand our contribution, we must review some basic aspects of CMASA.

#### Emulation and structural comparison in CMASA

**Figure 2:**
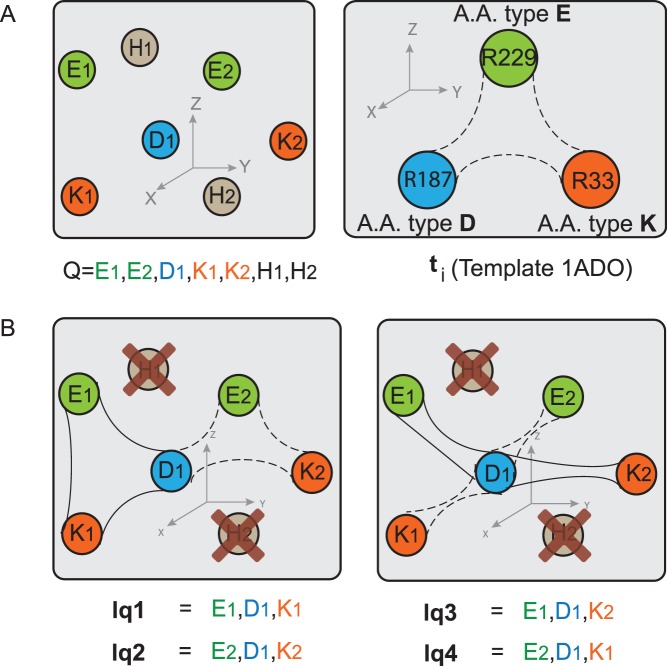
Emulation of local structures in CMASA. The query protein *Q* is hypothetical; the template *t_i_* is associated with the catalytic site of protein 1ADO to emulate the local structures. (A) Input to the method: *Q* is the sequence of residues in the query structure; *t_i_* (*E*, *D*, and *K*) is the template used to emulate the local structures in *Q*. (B) Emulated structures of *t_i_* in *Q*; there are four possible combinations (*lq_1_*, *lq_2_*, *lq_3_*, *lq_4_*) that may match the template *t_i_*.

Given a structural template of residues *t_i_* and a query structure *Q*, CMASA selects the residues in *Q* that match residues in *t_i_*. The local structures of *Q* are made of filtered residues. For instance, the example in Figure 2(A) presents a query protein with residues *E_1_, E_2_, D_1_, K_1_, K_2_, H_1_, H_2_* and the template *t_i_* with residues *E, D, K*. Then, filtering will eliminate residues *H_1_* and *H_2_*, and the remaining residues will be retained. Each possible combination of the remaining residues is compared with that in *t_i_*; there are a total of four local structures, *lq_1_* to *lq_4_*, as illustrated in Figure 2(B). If the pairwise distances are equivalent in both the local structure in the query (*lq_j_*) and in the template *t_i_*, a tentative match is found. The pairwise distances, i.e. the distance in the query and the distance in the template, are equivalent if the difference between them is less than or equal to the CMAD cutoff. The described procedure is repeated for every template *t_i_* in the database to determine the putative catalytic residues in *Q*. The matches are defined by those structures that are geometrically similar to a given template while the match itself has a low probability of being produced at random, i.e., lower than a p-value threshold.

**Figure 3:**
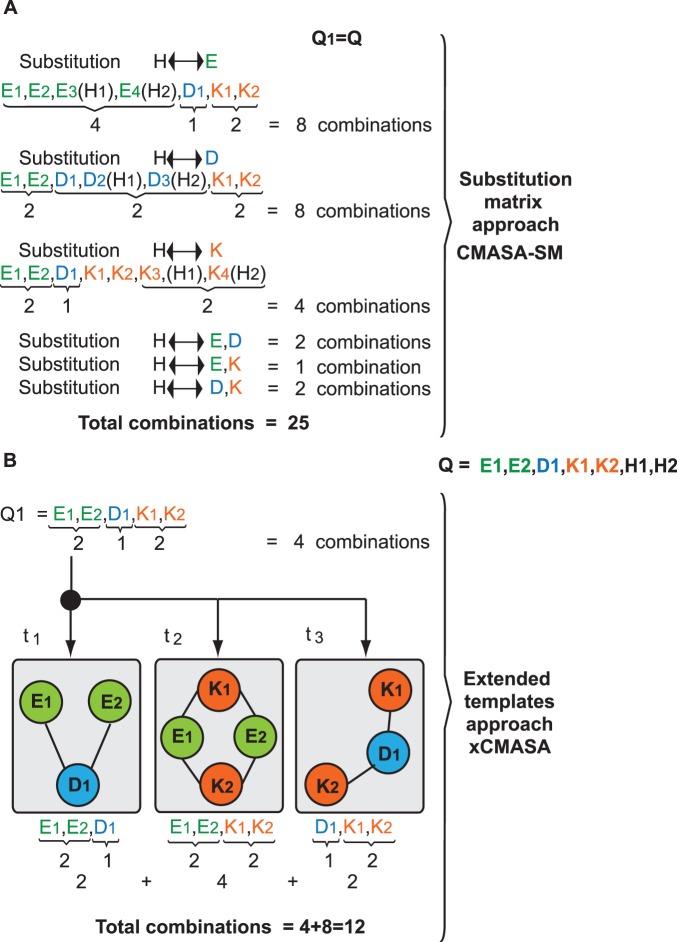
Computing the number of comparisons to handle single point mutations. Using the input shown in Figure 2 and the substitution matrix approach, the residues of type H can be interchanged with E, D, and K. These substitutions generate the combinations shown in (A). In contrast, xCMASA does not require additional information because the sub-templates are derived from *t_i_*, as shown in (B).

When the query protein has a hidden template *t_i_* but also has a single point mutation, CMASA proposes the use of a Substitution Matrix (SM) *S* in a scheme that we term CMASA-SM. S_i,j_ is 1 if an amino acid of type *i* is interchangeable with that of type *j*, where *i, j* ∈ [0, 19] represent the 20 amino acids. One of the disadvantages of this approach has to do with the number of comparisons that must be performed. For instance, let us assume that we have the query shown in Figure 2. After the filtering process, the following amino acids remain: *E_1_*, *E_2_*, *D_1_*, *K_1_* and *K_2_*. Let us assume that SM (*S*) will account for substitutions of the following type: *H*↔*E*, *H*↔*D*, and *H*↔*K*. Thus, in the first substitution, *H_1_* and *H_2_* in the query can be replaced by *E*; therefore, we have four *E*s to combine with one *D* and two *K*s. This substitution thus adds 8 combinations. If we follow the same analysis, we will show that the *H*↔*D* replacement adds also 8 new combinations, and the *H*↔*K* substitution adds 4 new combinations. In the SM approach, we can also consider simultaneous substitutions, such as *H*↔[*E,D*], *H*↔[*E,K*] and *H*↔[*D,K*]; these substitutions will add 2, 1, and 2 new combinations, respectively. The total number of combinations, as shown in Figure 3 (A), will be 25. However, the use of extended templates results in 4 original combinations plus 8 new combinations added by the subtemplates. In Figure 3 (B), every possible combination of two residues that will be a search term is indicated by an edge that connects two nodes.

#### CMASA and mutated or missing residues

CMASA fails to predict catalytic sites if the following conditions occur: *i)* there is a difference in the number of residues between the template and the query, i.e., the template has more residues than the query; *ii)* the *fa* atom is missing in any catalytic residue of the query; *iii)* there is a difference in the type of residues between the template and the query. The latter implies that mutated sites will not be recognized by CMASA; except in the case they are annotated in the database. When the number of residues in the template *t_i_* is smaller than the number of residues in catalytic site of the query, CMASA will produce correct matches. However, if the number of residues in *t_i_* is larger than in the number of residues in the query pattern, CMASA will not produce a correct match. The proposed extension is aimed at improving the prediction when any of these situations occur. Although CMASA-SM offers a solution to case *iii*), it cannot handle cases *i*) and *ii*).

In a previous paper [34], the site-directed mutagenesis of disulfide isomerase (EC 5.3.4.1) was analyzed. The catalytic site of this enzyme is composed of C-G-H-C; when this site is mutated to S-G-H-C, the enzyme retains one of its functions. Similar cases where one of the residues undergoes a mutation will not be recognized by CMASA using an identity SM. As a proof of concept, to show that CMASA may fail in the presence of single point mutations, we mutated a catalytic residue in 1MEK (EC 5.3.4.1), which has a catalytic site of C36-G37-H38-C39; the *in silico* mutagenesis of C36S was performed with Swiss-PdbViewer [35]. With this variant, CMASA is unable to predict the catalytic residues or even to associate the mutant with any enzymatic class. CMASA-SM can determine the site when every possible mutation of C to a polar residue is accounted for in the applied SM. However, if the SM accounts for changes of C to any other amino acid, the CMASA-SM software does not return any significant putative site because the abundance of residues increases, and the p-value becomes larger than the threshold. Furthermore, as we will show later, there may be cases where even if we tell CMASA-SM the specific residue to mutate, the software can still output a wrong result.

Perhaps the strongest limitation of CMASA is the use of the substitution matrix mechanism to address mutations. This mechanism is limiting for the following reasons: given a query, the possible substitutes are not known beforehand; therefore, SM should be applied to the template and not to the query. If we apply an SM that is based on the template, then each catalytic residue in *t_i_* should be dealt with separately to minimize the computational cost. However, even if we deal with the residues of *t_i_* one by one, the number of comparisons will be large. As an illustrative example, in Figure 3, the catalytic residues *E*, *D*, *K* are allowed to mutate to *H*, i.e., a single interchange per catalytic residue. However, in a real case, each catalytic residue will be substituted by a set of equivalent residues, i.e., every catalytic residue will contribute a particular SM, which will increase the number of comparisons. Another limit of CMASA-SM is that the abundance of residues will increase the p-value, as indicated by equations 3 and 4.

Although CMASA-SM is able to recover mutated and non-mutated residues, xCMASA recovers only the non-mutated ones.

#### Extended CMASA: xCMASA

The limitation introduced by the SM can be overcome. One method starts from each template *t_i_* of *n_ti_* residues and generates all subtemplates of *n_ti_*-1 residues; these subtemplates are used to extend the library of templates. This idea can be extended to generate all subtemplates of *n_ti_*-*k* residues; however, as *k* approaches *n_ti_*/2, the number of subtemplates increases exponentially. Fortunately, single point mutations (*k* = 1) will be the most abundant for a biological point of view. Another option can be to check for the subtemplates when the local structures of the query are compared with the templates. Due to the implementation simplicity, we choose the first option; however, both options are equivalent. The details for the selected option are given next.

The proposed extension includes three main components:

1. *Generation of new templates from the ones in T.* Given a template *t_i_*, the number of new templates will be 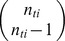 because each new template is a combination of *n_ti_* - 1 residues. That is, from each template *t_i_*, *n_ti_* new subtemplates will be generated and added to the library. The exclusion of a single residue from *t_i_* allows the recovery of the non-mutated residues of *t_i_*. If *n_max_* is the maximum number of residues that a template may have and there are *N_T_* templates, then the size of the library of templates will be increased by, at most, *N_T_*×*n_max_* elements.

In case of the SM, a single replacement strongly affects the number of combinations with residues to form local structures, as shown in the example of Figure 3.

2. *CMAD Tuning.* Adding smaller templates (fewer catalytic residues) increases the chances of random matches between the subtemplates and the local structures of the query. Tuning the threshold for CMAD can overcome this problem. This tuning will be described in the Results and Discussion section.
3. *Postprocessing.* The matches involving extended templates are filtered when the original template from which they were derived is also present in the match. The only goal of this part is to avoid excessive redundant information in the output file, and this step does not affect the efficiency of the proposed algorithm.

The implementation of xCMASA is based on the CMASA code, which is publicly available at http://159.226.149.45/other1/CMASA/CMASA.htm, under a GNU license.

The authors will provide xCMASA upon request.

## Results and Discussion

### Design of Computational Experiments

The computational experiments are divided into four scenarios. The first scenario is aimed at reproducing the results reported in [29]. The second scenario assesses the predictions obtained by xCMASA in the case when there are not mutations. The third scenario assesses the CMASA performance when a single point mutation is applied to the catalytic sites. The fourth scenario analyzes the performance of xCMASA when mutated proteins are the queries for the method. All scenarios were evaluated with a default CMAD value of 1.2.

#### 1. NoM_MT (No Mutation and Master Templates) scenario

This scenario used the test set A, which is described in the Materials and Methods section. The experiments presented in [29] were reproduced, and the results are shown in Table 1. We can observe an improvement over the results obtained in [29], mainly because we performed filtering of the test instances. The filtering eliminates some instances where CMASA fails.

**Table 1:** Replication from [29] for master templates and non-mutated queries.

Case	Sn	Acc	Pr	MCC
CMASA [29]	0.750	0.940	NA	0.820
Replication	0.795	0.981	0.907	0.840

#### 2. M_MT (Mutation and Master Templates) scenario

This scenario used the test set B and is set to evaluate the performance of CMASA when mutated proteins are introduced as queries. As shown in Table 2, with the exception of accuracy, the other performance criteria decreased considerably (M_MT) in this scenario relative to those from the previous scenario (NoM_MT). Notice that although the accuracy does not decrease, the method can only identify 3 proteins out of 744; these proteins matched with smaller master templates from different families. To address mutations, CMASA proposes the use of SM. To analyze the limitations of this approach, we select a set of ten instances, which are shown in Table 3. In all of these cases, we used an SM with the knowledge that the residues will undergo a single point mutation. Even in this case, CMASA-SM only produced matches that were above the p-value threshold. In three cases (1AHP, 1B4K, and 1G8F), no matches were generated. With the exception of these three cases, all others, except 1A3H, are the first matches that have a p-value that is above the threshold. One can think that a solution to this problem will be to change the p-value threshold; however, the correct match for 1A3H appears in the 6th position; therefore, tuning the p-value will not solve these types of cases. More importantly, increasing the p-value threshold will increase the number of *FP*s for the cases with no mutations.

**Table 2:** Performance criteria for CMASA (MT) and xCMASA (ET) with CMAD = 1.2. Mutated (M) and non-mutated (NoM) queries.

Scenario	TP	FN	FP	TN	Sn	Acc	Pr	MCC
NoM_MT	592	152	60	10515	0.795	0.981	0.907	0.840
NoM_ET	592	152	237	10338	0.796	0.966	0.714	0.736
M_MT	3	477	60	10515	0.006	0.951	0.048	0.001
M_ET	357	123	237	10338	0.744	0.967	0.601	0.652

**Table 3:** Examples of the predictions for catalytic sites that were evaluated as FNs by CMASA-SM and as TPs by xCMASA.

Protein	Master template	Catalytic site	Mutation by alanine scanning	p-value CMASA-SM	p-value xCMASA
1AHP	1A8I	K533-R534-K539-T641	R534A	NA	7.377E-06
2H12	1AL6	S242-H272-H313-D371	H272A	1.245E-03	3.544E-09
1B4K	1AW5	D127-S175-K205-K260	K205A	NA	8.003E-06
1CSN	1CKI	D131-K133-D135-N136-T181	K133A	1.326E-03	1.460E-09
1BHE	1CZF	D202-D223-D224-H251	H251A	3.079E-03	4.088E-06
1KKT	1DL2	E122-R126-D267-E409	E122A	2.100E-03	7.518E-09
1A3H	1EDG	N138-E139-H200-Y202-E228	Y202A	2.724E-01	2.680E-05
1F6D	1F6D	D95-E117-E131-H213	E117A	6.296E-03	1.736E-05
1G8F	1I2D	R197-H201-H204-R290	R290A	NA	4.555E-05
1BS4	1LME	G45-Q50-L91-E133	Q50A	1.060E-03	2.132E-08

#### 3. NoM_ET (No Mutation and Extended Templates) scenario

This scenario used test set A, which is generated using the extended templates, i.e., 744 proteins from the positive group and 10575 from the negative group. The scenario was proposed to assess xCMASA using non-mutated query structures. The results of this scenario differ from the results obtained using the NoM_MT scenario and the original CMASA because of the number of *FP*s, as shown in Table 2. Although CMASA has 60 *FP*s (NoM_MT and M_MT), xCMASA generates 237 *FP*s (NoM_ET and M_ET), mainly because the smaller extended templates increase the probability of generating random matches that are below the CMAD threshold. This situation can be improved by tuning the threshold for CMAD, as we will show shortly. It is interesting to note that several catalytic sites were partially identified by xCMASA, e.g., the site for the 1AHP protein, even when CMASA classified the site as an *FN* (Table 3).

#### 4. M_ET (Mutation and Extended Templates) scenario

This scenario used the test set B, and its goal was to assess the performance of the proposed extension when the queries are entirely composed of mutated proteins; only single point mutations are considered. The obtained results were 0.967 accuracy, 0.744 sensitivity, and 0.652 MCC (Table 2 (M_ET)). Notice that the main difference with the performance using test set A is that the proportion of *FN*s generated by xCMASA is larger using this test set.

**Figure 4:**
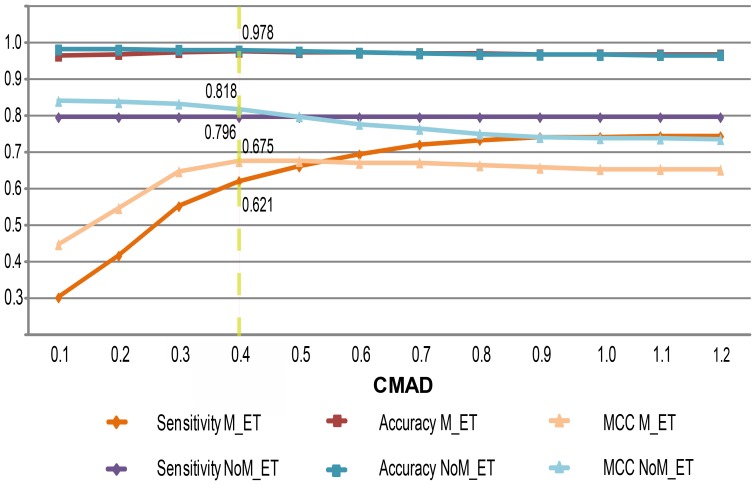
Performance measures as a function of CMAD. The graph shows the relations among the sensitivity, accuracy, and MCC of CMASA and xCMASA for scenarios with and without single point mutations.

The second and fourth scenarios demonstrated that xCMASA produced a larger number of *FP*s than CMASA did. This is tailored to the new added templates. The smaller templates increase the coincidences with local structures while querying proteins in the negative group (i.e., the proteins that do not have a catalytic function). One way to decrease this effect is to reduce the CMAD threshold. However, small CMAD values increase the number of false negatives because the mutated local structures are discriminated (see the sensitivity curve for scenario M_ET in Figure 4). A CMAD value of 0.4 is selected as a good tradeoff between not too many *FP*s for non-mutated instances and too many *FN*s for the mutated cases. In addition, CMAD = 0.4 gives the highest value for the sum of all performance criteria for xCMASA when using test set B. Table 4 shows the performance measures for xCMASA using mutated and non-mutated instances and a CMAD value of 0.4.

**Table 4:** Criteria and performance metrics using xCMASA with CMAD = 0.4. Mutated (M) and non-mutated (NoM) queries.

Scenario	TP	FN	FP	TN	Sn	Acc	Pr	MCC
NoM_ET	592	152	93	10482	0.795	0.978	0.864	0.818
M_ET	298	182	93	10482	0.621	0.975	0.762	0.675

The sensitivity of CMASA varies as a function of the CMAD threshold, i.e., if we decrease the cutoff to a value below 1.2, the sensitivity decreases. For instance, the sensitivity is 0.795 for a 1.2 CMAD cutoff, 0.718 for a 0.4 cutoff, and 0.601 for a 0.1 cutoff; however, the sensitivity of xCMASA does not change as a function of the CMAD threshold for non-mutated instances (as shown in Figure 4). This behavior occurs because the templates in xCMASA are smaller. Thus, whenever a match is found between these extended templates and the input structures, the computed CMAD values are less than 0.1. Therefore, xCMASA has the same sensitivity for all CMAD cutoff values, i.e., values from 1.2 to 0.1.

In addition to the alanine scanning approach to evaluate the extension, the performance for a set of real cases was also analyzed. In this scenario, two study cases were proposed.

### Study Cases

To select each of these cases, the following procedure was performed. First, a literature review identified a family of mutated proteins that have a small number of representatives in CMASA (CMASA includes 15341 templates). Second, some proteins with mutation in the catalytic site of interest were identified from a structural comparative analysis with different elements in the family. The web service PDBeFold [36] was used for this analysis. The study cases were designed to evaluate the capability of the original CMASA, CMASA-SM, and xCMASA of identifying the catalytic residues in the following situations: a protein with actual mutations or a protein whose PDB entry is missing relevant information.

**Figure 5:**
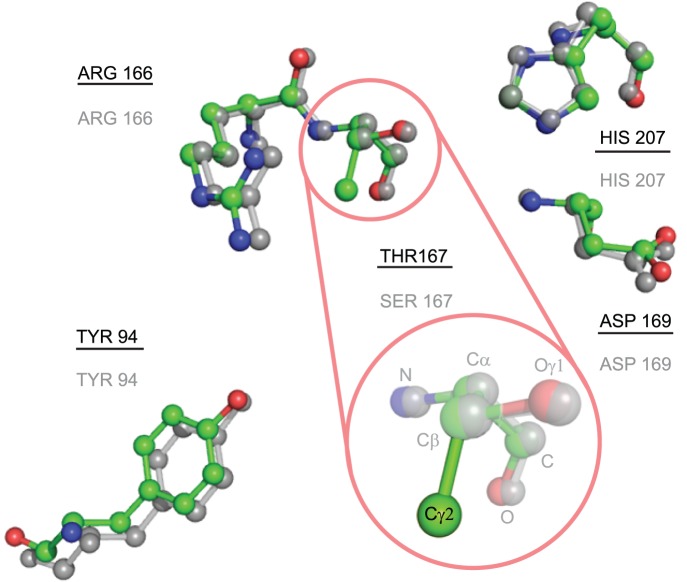
Comparison of local catalytic structures in 1EVF and 1BQ1. Residue number 167 in 1EVF is mutated from SER to THR, and the proposed extension could detect all of the remaining catalytic residues, which were not detected by CMASA or CMASA-SM. Superposition of the catalytic residues in the query 1EVF (colors by element with the identifiers underlined) and the residues of the associated templates 1BQ1 (the elements are in grey, and the identifiers are not underlined). The similarity of the local structures of the non-mutated residues is shown.

**Figure 6:**
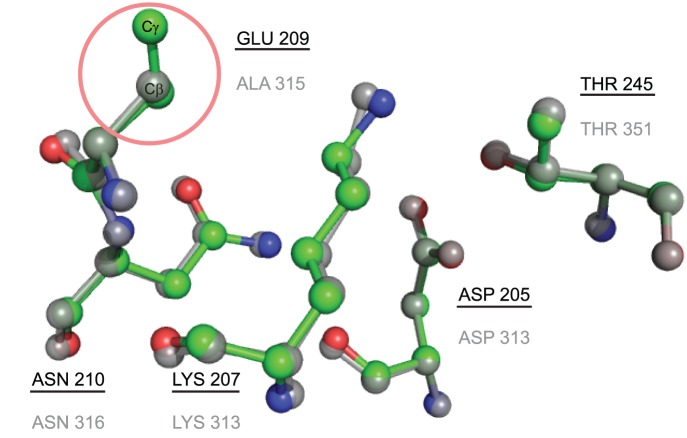
Comparison of local catalytic structures in 3HRC and 2OIC. The template of 2OIC has a catalytic site structure similar to that of 3HRC, with the exception of residue A315. The proposed xCMASA is able to detect the catalytic residues D205-K207-N210-T245. Superposition of the catalytic residues in the query structure 3HRC (the colors differ for each element, and the identifiers are underlined) and the residues of the associated 20IC (the elements are in grey, and the identifiers are not underlined).

- The role of residue S167 in the catalytic function of *Escherichia coli* thymidylate synthase (EC 2.1.1.45) was analyzed previously [37]. The structure 1EVF contains the variant S167T (see Figure 5), and the annotated catalytic residues in CSA for this protein are E58-W80-Y94-C146-R166-D169-N177. This protein does not have a template in the CMASA database, although there are a series of thymidylate synthase mutants at the serine 167 residue (1EV5 corresponds to S167A and 1EVG corresponds to S167T). For both, the catalytic residues are Y94-C146-R166-D169-N177, which have the same residues as in 1EVF. However, because of the change in serine 167, CMASA cannot detect the other catalytic residues that remain unchanged: the program outputs no matches below the p-value threshold, and the matches over the threshold do not belong to the same family of enzymes. CMASA-SM was tested for the case in which SM accounts for all possible substitutions of *S* by polar residues, i.e., S↔[*T*, *Y*, *H*, *C*, *N*, *Q*, *W*]. The first match identified by CMASA-SM is 1QQQ, which has the putative residues E58-T167-D169-N177-H207. In this case, T167 and H207 are not annotated residues. Notice that this is an optimistic scenario for CMASA-SM because the proper substitution matrix is not known beforehand for a query protein, in this case 1EVF. In contrast, xCMASA was able to determine the similarity even in this case, i.e., xCMASA identified the matching residues as Y94-R166-D169-N177, a sub-template of 1EV5; the p-value was 0.0, and the CMAD was 0.1 for this first hit.
- We analyzed 3HRC as our first example of missing data; this protein kinase domain belongs to the transferase family, it has D205-K207-E209-N210-T245 as catalytic residues, and its E.C. number is 2.7.11.1. The CMASA database has many templates that match 3HRC in terms of geometry, amino acid types and enzymatic classification. For instance, the structure of 2OIC has the following catalytic residues: D311-K313-A315-N316-T351. Similarly to 3HRC, 2OIC is annotated as a transferase with the EC number 2.7.11.1 and differs from 3HRC in the residues E209-A315 (see Figure 6). In this case, even if we provide CMASA-SM with the correct residue to mutate (*E* by *A*), CMASA-SM does not identify a match that is below the threshold because of the missing atom. In contrast, xCMASA was able to identify the residues D205-K207-N210-T245 with a p-value of 2.2×10^−16^ and a CMAD of 0.114, and the proteins associated with the prediction belong to the same enzymatic class.

**Figure 7:**
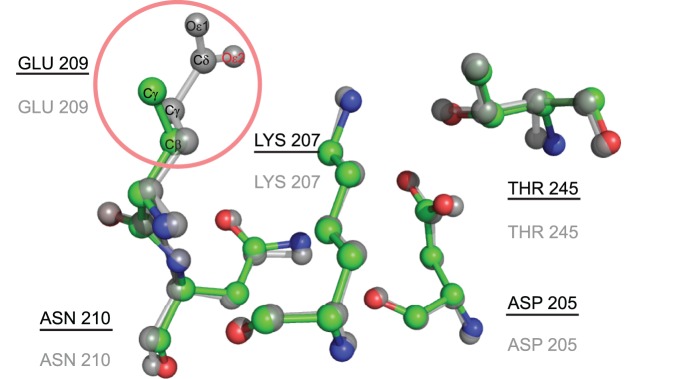
Comparison of local catalytic structures in 3HRC and 1UU9. Although the proteins 3HRC (the colors differ by element, and the identifiers are underlined) and 1UU9 have the variants with A209E (in grey, and the identifiers are not underlined), 3HRC lacks an atom in the side chain (*O*ε*_2_*). CMASA fails in this situation, but xCMASA is able to detect the non-mutated residues.

We also consider 3HRC again with the template of 1UU9 and the catalytic residues D205-K207-E209-N210-T245 for another example of missing data; the catalytic residues in 1UU9 are the same as those in 3HRC. However, CMASA fails to predict all residues because 3HRC does not contain the atom *O*ε*_2_* in residue E209 (see Figure 7). This is the furthest atom in the side chain for this residue; thus, the residue is not represented by the method, and the local structure emulation and evaluation processes disregarded all matching templates. In contrast, xCMASA lists 1UU9 as the third match (after 2OIC and 20IB) when 3HRC is the query. Incomplete information in a PDB entry is not a rare event, and this missing information can affect the prediction in CMASA or CMASA-SM. However, when an atom is missing, a non-trivial preprocessing of completing the PDB entry can solve the problem. In the described study case, an SM was used. However, CMASA halts after some computations because it cannot handle the large number of candidate matches. This number becomes large because every *E* in the query protein is replaced by the other 19 amino acids, and each of the 19 amino acids in the query is replaced by *E*.

### Conclusions

xCMASA has been proposed as an extension of the CMASA method. The extension is based on the generation of a set of sub-templates. The proposed variant allows CMASA to account for pointwise mutations in an efficient manner. xCMASA preserves the prediction power of CMASA in cases without mutations at an additional cost bounded by the product of the maximum number of residues among the templates times the number of templates. xCMASA overcomes the CMASA-SM limitation of having to provide an SM with the correct substitutions. The method not only simplifies the process of adjusting the substitution matrix but also identifies catalytic sites even when the furthest atom is missing in a single catalytic residue.

## Supporting Information

Table S1 **Positive group in test set A.** The table contains the proteins and the families that they belong to, used in the test set A of xCMASA.
(DOCX)

Table S2 **Set of mutated proteins by alanine scanning.** Information of the mutated proteins and its catalytic residues, these are the elements of the positive group in test set B of xCMASA.
(DOCX)
