# Supplementary material for: A Simple Extension to the CMASA Method for the Prediction of Catalytic Residues in the Presence of Single Point Mutations
Source: PLoS One. 2014 Sep 30;9(9):e108513. doi: 10.1371/journal.pone.0108513 (PMC4182483; doi:10.1371/journal.pone.0108513)
Supplement: Table S1 — Positive group in test set A. The table contains the proteins and the families that they belong to, used in the test set A of xCMASA. (DOCX) [file pone.0108513.s001.docx]

Table S1. **Positive group in test set A.** The table contains the proteins and the families that they belong to, used in the test set A of xCMASA.

| **Family name** | **Active sites** | **Family members** | **Excluded members** |
| --- | --- | --- | --- |
| 1A8IA | K568-R569-K574-T676 | 1A8IA*1AHPA*1EM6A*1GPAA*2C4MA |  |
| 1ACOA | D100-H101-H147-D165-H167-E262  (CMASA database has no D100) | 1ACOA*1FGHA | 2B3XA (mismatch in catalytic residues) |
| 1ADOA | D33-E187-K229 | 1ADOA*1A5CA*1ALDA*1EPXA*1F2JA*1FBAA*1FDJA *1XFBA |  |
| 1AFWA | C125-H375-C403-G405 | 1AFWA*2C7YA*2IIKA | 1WDKC(no record in CMASA database) |
| 1AJOA | E193-D195-E197 | 1AJOA*1AJKA*1BYHA*1CPMA*1GBGA*1MVEA*2AYHA |  |
| 1AL6A | S244-H274-H320-D375 | 1AL6A*1A59A*1K3PA*1O7XA*2H12A*2IFCA |  |
| 1AQUA | K48-H108-S138 | 1AQUA*1G3MA*1HY3A |  |
| 1AT1A | R54-T55-R105-H134 | 1AT1A*1ML4A*1PG5A*2BE7A |  |
| 1ATJA | R38-H42-N70 | 1ATJA*1FHFA*1PA2A*1SCHA*7ATJA | 1ARPA(no record in CMASA database) |
| 1AW5A | D131-S179-K210-K263 | 1AW5A*1B4EA*1B4KA*1E51A*1H7OA*1W1ZA |  |
| 1BDMA | D158-R161-N185-H186 | 1BDMA*1B8PA | 4MDHA(extra catalytic residue type S ) |
| 1BITA | H57-D102-G193-S195-G196-S214 | 1BITA*1AQ7A*1FN8A*1FXYA*2EEKA |  |
| 1BK7A | H34-E84-H88 | 1BK7A*1BOLA*1DIXA*1IQQA |  |
| 1BMFD | K162-E188-R189-R356 | 1BMFD*1OHHD(added) *1SKYE*2DPYA |  |
| 1BX4A | R132-G297-A298-G299-D300 | 1BX4A*1DGMA*1LIJA*1LIOA |  |
| 1BZCA | D181-C215-R221-S222 | 1BZCA*1L8KA*1WCHA*2QEPA |  |
| 1C2BA | S203-E334-H447 | 1C2BA*1ACJA*1DX4A*2ACEA |  |
| 1C9WA | D43-Y48-K77-H110 | 1C9WA*1AH0A*2BGQA |  |
| 1CBGA | E183-N324-E397 | 1CBGA*1GNXA*2RGLA |  |
| 1CBXA | R71-R127-E270 | 1CBXA*1ARLA*5CPAA |  |
| 1CF2O | C140-R167-H219 | 1CF2O*1B7GO*2YYYA |  |
| 1CGTA | H140-R227-D229-E257-F259-H327  (CMASA database has no F259) | 1CGTA | 1A47A*1CYGA (mismatch in catalytic residues) |
| 1CKIA | D131-K133-D135-N136-T181 | 1CKIA*1CSNA*1F3MC**2ESMA*2F2UA | 1T4HA (no record in CMASA database) |
| 1CLKA | H53-D56-E180-K182-H219-D254  (CMASA database has no D56) | 1CLKA*1A0CA*1A0DA*1A0EA | 1BXBA*1DIDA (mismatch in catalytic residues) |
| 1CMVA | H63-S132-H157-R165 | 1CMVA*1NKKA*1O6EA |  |
| 1CMXA | Q84-C90-H166-D181 | 1CMXA*1UCHA*2ETLA |  |
| 1CV2A | D108-W109-E132-H272 | 1CV2A*1BN6A*2O2HA |  |
| 1CVIA | R1011-H1012-R1015-R1079-H1257-D1258  (CMASA database has no R1015) | 1CVIA | 1RPAA*1RPTA (mismatch in catalytic residue type D) 2GLAA (no record in CMASA database) |
| 1CZFA | D180-D201-D202-H223 | 1CZFA*1BHEA*1IA5A*1NHCA*2IQ7A | 1K5CA (extra catalytic residue type S ) |
| 1D3AA | D168-R171-H195 | 1D3AA*1GUYA*1GUZA*1GV0A*1OJSA  *2D4AA | 1EMDA*1SEVA*1MLDA (extra catalytic residue type S ) |
| 1D3GA | F149-S215-T218-K255 | 1D3GA*1F76A*1TV5A |  |
| 1D6OA | D37-I56-Y82 | 1D6OA*1A7XA*1C9HA*1FD9A*1IX5A*1JVWA*1N1AA  *1PBKA*1Q6HA*1U79A*1YATA*2PBCA |  |
| 1DJLA | Y890-R925-Y1006 | 1DJLA*1D4OA*1E3TA | 2BRUC (no record in CMASA database) |
| 1DL2A | E132-R136-D275-E435 | 1DL2A*1FMIA*1KKTA*1NXCA |  |
| 1DNPA | W306-W359-W382 | 1DNPA*1IQRA*1OWLA |  |
| 1DV7A | K42-D70-K72-D75 | 1DV7A*1DBTA*1DQWA*1EIXA*1VQTA*2CZ5A*2F84A  *2YYTA |  |
| 1DXLA | C45-C50-H449-E454 (CMASA database has extra residue T125 ) | 1DXLA | 1EBDA*1JEHA*1ZMCA*3LADA (without amino acid type T). 1LPFA*1LVLA*2A8XA (mismatch in catalytic residues) |
| 1DYSA | Y86-R91-D92-D139-D316 | 1DYSA*1TMLA*1UOZA |  |
| 1DYWA | R62-F67-Q70-N109-F120-L129  (CMASA database has no F120) | 1DYWA*1A33A*1ISTA*1MZWA*1QNGA*1XWNA*2A2NA*  2BITX*2ESLA*2GW2A*2PLUA | 1AK4A*1CYNA*1H0PA*1IHGA**1M9CA*1W74A*1XYHA*1XO7A*1Z81A*1ZKCA*1ZMFA*2B71A*2CK1A*2FU0A*2HAQA*2HQ6A*2HQJA (mismatch in catalytic residues) |
| 1E1OA | R262-E278-R480 | 1E1OA*1BBUA*3BJUA |  |
| 1E7PA | H257-R301-H369-R404  (CMASA database also has E294) | 1E7PA | 1D4CA*1QO8A (extra catalytic residue in master template).  1KF6A*1NEKA (mismatch in catalytic residues) |
| 1EC7A | K205-K207-D313-H339-D366 | 1EC7A*1BQGA*1EC9A |  |
| 1ECLA | E9-D111-Y319-H365 | 1ECLA*1CY0A*2GAIA |  |
| 1EDGA | N169-E170-H254-Y256-E307 | 1EDGA*1A3HA*1CECA*1ECEA* |  |
| 1EG1A | E196-D198-E201-H212 | 1A39A*1EG1A*1OVWA |  |
| 1EH6A | N137-C145-H146-E172 | 1EH6A*1MGTA*1SFEA*1WRJA*2G7HA |  |
| 1EJJA | S62-D154-R261 | 1EJJA*1O98A*2IFYA |  |
| 1EMSA | Q379-H390-H392 | 1EMSA*1FHIA*5FITA |  |
| 1F6DD | D95-E117-E131-H213 | 1F66D*1F6DA*1V4VA*3BEOA |  |
| 1F75A | R33-R42-R197-R203 | 1F75A*1JP3A*2D2RA*2VG2A |  |
| 1FCQA | D111-E113-Y184-Y227-W301 | 1FCQA*2ATMA*2PE4A |  |
| 1FGXA | E317-D319-R359 | 1FGXA*1FR8A*1NF5B |  |
| 1FJMA | D95-R96-N124-H125-R221-H248  (CMASA database has no D95) | 1FJMA*1IT6A*1S70A*1S95A | 1AUIA (mismatch in catalytic residues) |
| 1FOBA | R45-E136-E246 | 1FOBA*1FHLA*1HJQA*1HJSA |  |
| 1FUGA | H14-K165-R244-K245-K265-K269  (CMASA database has no K265-K269)  (CMASA database also has D271) | 1FUGA*1O90A*2P02A |  |
| 1FUOA | T187-H188-S318-K324-E331 | 1FUOA*1VDKA*1YFMA |  |
| 1FUSA | H40-E58-H92 | 1FUSA*1B2MA*1RDSA |  |
| 1FX0A | K176-Q201-K202-R366 | 1FX0A*1SKYB*2R9VA |  |
| 1G0DA | C272-H332-D355-Y515 | 1G0DA*1EVUA*1KV3A*1L9MA |  |
| 1G0ZA | G30-H48-D94 | 1G0ZA*1A2AA*1A3DA (ADDED) | 1AE7A*1AOKB*1AYPA  *1B4WA*1BK9A*1BP2A*1BUNA*1CL5A*1DPYA*1FX9A*1G2XA*1GMZA*1GP7A*1IJLA*1LE6A*1M8TA*1MH2A*1MH2B*1MH7A*1N29A*1OQSB*1OWSA*1OWSB*1OZ6A*1OZYA*1PO8A*1PP2R*1PWOA*1VAPA*1VIPA*2OQDA*2QHDA (no record in CMASA database) |
| 1G2OA | H90-E93-N231 | 1G2OA*1C3XA*1M73E*1TCUA*1ULAA*2P4SA |  |
| 1GALA | E412-H516-H559 | 1GALA*1CF3A*1GPEA |  |
| 1GEHA | K163-K165-K189-D191-H281-H314  (CMASA database has no H314) | 1GEHA*2CWXA | 1BXNA*1SVDA (mismatch in catalytic residues) |
| 1GERA | C42-C47-K50-Y177-E181-H439  (CMASA database has no C47) | 1GERA*1GETB*1ONFA |  |
| 1GK8A | K175-K177-D203-H294-H327 | 1GK8A*1BWVA*1RBLA | 1AA1L (no record in CMASA database) |
| 1GOKA | E131-H209-E237-D239 | 1GOKA*1B30A*1CLXA*1E0VA*1HIZA*1NQ6A*1TA3B*1XYZA*2F8QA |  |
| 1GPIA | E207-D209-E212-H223 | 1GPIA*1CELA*1Q9HA |  |
| 1GQNA | E86-H143-K170 | 1GQNA*1QFEA*2EGZA*2OCZA*2YR1A |  |
| 1GX3A | C70-H110-D127 | 1GX3A*2BSZA*2IJAA*2PFRA*2VFBA |  |
| 1GZ7A | S209-E341-H449 | 1GZ7A*1CLEA*1CRLA |  |
| 1HVXA | H106-R232-D234-E264-W266-D269  (CMASA database has no C47) | 1HVXA*2DIEA | 1HT6A (mismatch in catalytic residues) |
| 1I2DA | R199-H203-H206-R292 | 1I2DA*1G8FA*1JHDA |  |
| 1ITKA | R92-H96-D125-N126 | 1ITKA*1MWVA*1SJ2A*1UB2A |  |
| 1IYKA | N175-F176-L177-L451 | 1IYKA*1IICA*2NMTA |  |
| 1J3NA | C161-H301-K333-H338-F394-F396  (CMASA database has no F396) | 1J3NA*1B3NA*1DD8A*1E5MA*1OX0A*1W0IA*2GP6A |  |
| 1JCZA | H64-E106-T199 | 1JCZA*12CAA*1AZMA*1CA2A*1CA3A*1KOPA*1RJ5A *1V9EA*1ZNCA*2ZNCA |  |
| 1JNKA | D189-K191-S193-N194-T226 | 1JNKA*1A9UA*2B9FA |  |
| 1JS4A | D55-D58-E424 | 1JS4A*1CLCA*1G87A*1KS8A |  |
| 1K2RA | C415-R418-W587-E592 | 1K2RA*1D0CA*1NSIA*3NOSA |  |
| 1K4LA | D41-C66-Y94-D99-H136-E174  (CMASA database has no Y94) | 1K4LA*1K49A*1TKSA |  |
| 1KFWA | D188-D190-E192-Y271 | 1KFWA*1D2KA*1GUVA*1ITXA*1W9PA |  |
| 1KHBA | H264-K290-R405 | 1KHBA*1NHXA*2FAFA |  |
| 1L8XA | H235-H312-E314 | 1L8XA*1AK1A*1HRKA*2C8JA |  |
| 1LARA | E1428-D1490-H1521-C1522-R1528-T1529  (CMASA database has no T1529) | 1LARA | 1JLNA*1RPMA*1YFOA*1ZC0A*2AHSA*2B49A*2BZLA*2C7SA*2FH7A*2G59A*2H4VA*2OOQA*2P6XA  *2PA5A (mismatch in catalytic residues) |
| 1LMEA | G44-Q49-L88-E130 | 1LMEA*1BS4A*1BSJA*1IX1A*1LM4A*1LM6A*1LQYA*1SV2A*1V3YA*1WS0A*1ZXZA*2OKLA |  |
| 1M56A | H284-E286-Y288-K362-H419-F420  (CMASA database has H284-H419-H421-R481-R482) | 1M56A*1AR1A*1OCCA |  |
| 1MGRA | E57-R68-H88 | 1MGRA*1AY7A*1C54A |  |
| 1MO0A | N10-K12-H94-E164-G170 | 1MO0A*1AMKA*1AW1A*1BTMA*1CI1A*1HG3A*1HTIA  *1I45A*1LYXA*1M6JA*1SPQA*1TMHA*1W0MA*1YYAA  *2DP3A*2I9EA*2JGQA*2VEIA |  |
| 1MZHA | D188-K250-K279 | 1MZHA*1N7KA*1O0YA*1P1XA*1VCVA |  |
| 1N9BA | S70-K73-S130-E166 | 1N9BA*1ALQA*1AXBA*1BLCA*1BSGA*1BTLA*1BUEA  *1E25A*1G68A*1HZOA*1I2SA*2CC1A*2GDNA |  |
| 1NDBA | Y107-P120-H343-S554 | 1NDBA*1NDIA*1T7NA |  |
| 1NIDA | F64-G66-D98-H255 | 1NIDA*1AQ8A*1KCBA*1MZYA |  |
| 1NMWA | H59-C113-H157 | 1NMWA*1F8AB*1J6YA |  |
| 1O86A | H353-A354-E384-H513-Y523 | 1O86A*1J36A*1O8AA |  |
| 1OEPA | E165-H188-E208-K343-H371-K394  (CMASA database has no H188) | 1OEPA*1IYXA*1PDYA*1W6TA |  |
| 1OJ4A | K10-D141-T181 | 1OJ4A*1UEKA*2V2QA |  |
| 1ONRA | D17-E96-K132 | 1ONRA*1F05A*1I2NA*1VPXA |  |
| 1P5HA | Q17-E140-D169-G260-G261 | 1P5HA*1PQYA*1T4CA |  |
| 1PEMA | C178-N386-C388-E390-C415 | 1PEMA*1ZYZA*3R1RA |  |
| 1PFKA | G11-R72-T125-D127-R171 | 1PFKA*1MTOA*1ZXXA*2HIGA |  |
| 1QHFA | H8-R59-E86-H181 | 1QHFA*1T8PA*1YFKA | 1BQ3D (no record in CMASA database) |
| 1QK1A | R127-E227-R231-R287-R315 | 1QK1A*1CRKA*1G0WA*1I0EA*1VRPA*2GL6A |  |
| 1QPNA | R105-K140-E201-D222 | 1QPNA*1QAPA*1QPRA*2B7NA*2JBMA |  |
| 1QSGA | Y156-M159-K163 | 1QSGA*1C14A*1CWUA*1MFPA*2PD3A | 2P91A (extra catalytic residue ) |
| 1QWLA | H56-S95-N129 | 1QWLA*1A4EA*1CF9A*1DGBA*1E93A*1GWEA*1IPHA  *1M7SA*1SI8A*1SY7A*2ISAA*2IUFA*2J2MA |  |
| 1QWOA | R58-H59-R62-R142-H338-D339  (CMASA database has no R58-H59) | 1QWOA | 1IHPA*1QFXA (mismatch in catalytic residues)  2GFIA (extra catalytic residue ) |
| 1R1DA | S93-D192-H222 | 1R1DA*2C7BA*2O7RA | 1M33A (extra catalytic residue) |
| 1RP1A | F77-L153-D176-H263 | 1RP1A*1BU8A*1ETHA*1GPLA*1HPLA*1LPAB*2OXEA  *2PPLA |  |
| 1S1MA | C379-H515-E517 | 1S1MA*1VCMA*2VKTA |  |
| 1S57A | K91-Y131-N194 | 1S57A*1B4SA*1BE4A*1EHWA*1K44A*1NDLA*1NSPA*1PAEX*1PKUA*1U8WA*1W7WA*1WKJA*1XIQA*1XQIA*1ZS6A  *2AZ1A*2B8PA*2CWKA*2HURA | 1NHKR (no record in CMASA database) |
| 1T2AA | T155-E157-Y179-K183 | 1T2AA*1RPNA | 1DB3A (extra catalytic residue type R) |
| 1V8BA | H54-D134-K230-D234-N235-C239  (CMASA database has no N235-C239)  (CMASA database also has H345) | 1V8BA*1A7AA*1B3RA |  |
| 1VC4A | E51-K53-K112-E160-N181-S215 | 1VC4A*1A53A | 1I4NA (mismatch in catalytic residues) |
| 1VDCA | C135-C138-D139 | 1VDCA*1CL0A*1TDEA*2A87A*2Q0KA |  |
| 1VLCA | Y141-K187-D219 | 1VLCA*1A05A*1CM7A*1DPZA*1V53A*1W0DA*1WPWA |  |
| 1WE1A | H17-Y50-T126-R127-G130-D131  (CMASA database has no Y50-R127-G130)  (CMASA database also has G134-G135) | 1WE1A*1DVEA*1N3UA*1WOVA*2Q32A |  |
| 1WL4A | H353-C383-G385 | 1WL4A*2F2SA | 1QFLA true catalytic site:  L89, G348, L378 |
| 1WO8A | H9-G56-D61-D81-H88-D91  (CMASA database has no D61) | 1WO8A*1B93A*1VMDA |  |
| 1XG2A | Q109-Q131-D132-D153 | 1XG2A*1GQ8A*1QJVA |  |
| 1Y8GA | D175-K177-E179-N180 | 1Y8GA*1KWPA*2H34A*2H6DA*2HW6A | 2EUEA (no record in database) |
| 1YISA | T154-H155-K291-E298 | 1YISA*1C3CA*2J91A*3BHGA |  |
| 1YRPA | D139-K141-E143-N144-T180 | 1YRPA*1GZKA*1H1WA*1HOWA*1IA8A*1IG1A*1VZOA*1WAKA*1WMKA*1XQZA*2BFXA*  2C47A*2CHLA*2CMWA*2CN5A*2IWIA*2PZIA |  |
| 1YTMA | H225-K248-R327 | 1YTMA*1AQ2A*1II2A*1J3BA*1YGGA |  |
| 1Z4RA | F568-F573-E575-I576-V577-C579  (CMASA database has no F568-F573)  (CMASA database also has I642-Y645) | 1Z4RA*1CM0A | 1YGHA (mismatch in catalytic residues) |
| 1Z7WA | K46-S269-S297 | 1Z7WA*1OASA*2EGUA |  |
| 1ZIOA | K13-R127-R160-D162-D163-R171  (CMASA database has no R160) | 1ZIOA*1AKEA*1P3JA*1P4SA*1S3GA*1ZINA*2RGXA | 1AK2A*1AKYA*1Z83A (mismatch in catalytic residues) |
| 1ZUWA | D10-S11-C74-C185 | 1ZUWA*1B73A*2JFNA*2JFOA*2JFQA*2JFUA*2JFXA  *2OHGA |  |
| 2A6PA | H13-R64-E85-H147 | 2A6PA*1FZTA*1RIIA |  |
| 2AAZA | E79-C187-S217-D219-D255-H257  (CMASA database has no E79) | 2AAZA*1CI7A | 1LCBA (mismatch in catalytic residues) |
| 2B7AA | D976-A978-R980-N981 | 2B7AA*1BYGA*1K2PA*1MP8A*1SM2A*1U46A*1XBAA*1YVJA*3BKBA |  |
| 2BLTA/1BLSA | S64-K67-Y150-E272-K315 | 1BLSA(New master template) *1C3BA*1ZKJA | 2BLTA (No record found in CSA web server) |
| 2C07A | N171-S199-Q209-Y212-K216 | 2C07A*1EDOA*2P68A | 1UZLA (No record in CSA web server) |
| 2C2NA | S117-H234-Q283 | 2C2NA*1MLAA*2QC3A |  |
| 2DQ7X | D130-R132-A134-N135 | 2DQ7X*1AD5A*1FMKA*1QPCA |  |
| 2EBDA | C111-F156-H236-N266 | 2EBDA*1EBLA*1HZPA*1MZJA*1ZOWA |  |
| 2EHHA | T43-T44-Y132-R137-K161 | 2EHHA*1XXXA*2RFGA*2YXGA |  |
| 2EKCA | E49-D60-Y175 | 2EKCA*1A50A*1GEQA |  |
| 2EP5A | C151-Q178-H244 | 2EP5A*1BRMA*1NWCA*1YS4A*2GYYA |  |
| 2EWDA | D168-R171-H195 | 2EWDA*1A5ZA*1CEQA*1EZ4A*1I0ZA*1I10A*1LDBA*1LDMA*1LLCA*1LLDA*1PZEA*1SOVA*1V6AA*2E37A*2LDXA  *2V65A*2V6BA*3LDHA |  |
| 2F57A | D568-K570-D572-S573-T606 | 2F57A*2BVAA*2C30A |  |
| 2FEKA | C9-C14-R15-D115 | 2FEKA*1U2PA*1ZGGA |  |
| 2GBCA | S631-D709-D710-H741 | 2GBCA*1J2EA*1ORVA*2ECFA |  |
| 2GFOA | N781-C786-H1067-D1084 | 2GFOA*1NB8A*1NBFA*2AYNA |  |
| 2GSAA | Y150-D245-K273 | 2GSAA | 2CFBA*2E7UA (mismatch in catalytic residues) |
| 2HB6A | D257-K264-R338 | 2HB6A*1BLLE*1GYTA |  |
| 2HCYA | H44-T45-H48-W54 | 2HCYA*1H2BA*1RJWA |  |
| 2HFSA | K18-D155-T198 | 1KVKA*2HFSA*2OI2A | Deleted because no information avaible in CMASA database |
| 2HIHA | S124-D314-H355 | 2HIHA*1EX9A*1HLGA | 1TAHA (extra catalytic residues) |
| 2HS6A | H185-H188-Y190 | 2HS6A*1ICPA*1Q45A |  |
| 2I6UA | R52-T53-R101-H128-Q131-D224  (CMASA database has no D224) | 2I6UA*1A1SA*1C9YA*1DXHA*1VLVA |  |
| 2IV0A | Y156-K223-D277 | 2IV0A*1AI2A*1HQSA*1TYOA*2D1CA*2D4VA |  |
| 2NACA | N146-R284-Q313-H332 | 2NACA*2FSSA*2GO1A*2GSDA |  |
| 2OHCA | Y229-H236-K265 | 2OHCA*1A79A*1R0VA |  |
| 2OK7A | Y103-S104-C284-E314 | 2OK7A*1A8PA*1QFYA*2B5OA |  |
| 2PA6A | E170-H193-E213-K235-K339-H367  (CMASA database has no H193- K235)  (CMASA database also has K390) | 2PA6A*1E9IA*1EBGA |  |
| 2PGDA | G130-K183-N187-E190 | 2PGDA*1PGJA*1PGNA*2IYOA*2P4QA |  |
| 2Q3EA | T131-E165-K220-N224-C276-D280  (CMASA database has no C276) | 2Q3EA*1DLIA*2O3JA |  |
| 2Q8NA | K137-E143-G197-R198-E281-H310  (CMASA database has no E143-G197 )  (CMASA database also has K422) | 2Q8NA*1B0ZA*1DQRA*1Q50A*1U0EA*1ZZGA*2O2CA |  |
| 2QFLA | D44-E67-T89-D212 | 2QFLA*2BJIA*2CZHA*2Q74A |  |
| 2QT6A | H451-C452-H453 | 2QT6A*1A65A*1GW0A*1GYCA*1KYAA*1V10A*2H5UA  *2HZHA |  |
| 2RD5A | K41-G44-G77-K255 | 2RD5A*1GS5A*1OH9A*2AP9A*2BTYA*2BUFA |  |
| 2REIA | D758-A760-R762-N763 | 2REIA*1FVRA*1GAGA*1IR3A*1JPAA*1JQHA*1LUFA*1M14A*1MQBA*1PKGA*1R0PA*  1RJBA*1VR2A*1Y6AA*2GSFA*2HELA*2I0VA*2IVSA*2P0CA*2R2PA | 1AGWA(no record in CMASA database) |
| 2VGBA | R116-R163-K313-T371-S405-E407  (CMASA database has no R163) | 2VGBA*1A3WA*1A49A*1E0TA*1PKLA*1PKNA*2E28A |  |
| 2YXXA | K46-K153-H179-E246 | 2YXXA | 1KNWA*2P3EA (extra catalytic residues) |
