# Supplementary material for: A Simple Extension to the CMASA Method for the Prediction of Catalytic Residues in the Presence of Single Point Mutations
Source: PLoS One. 2014 Sep 30;9(9):e108513. doi: 10.1371/journal.pone.0108513 (PMC4182483; doi:10.1371/journal.pone.0108513)
Supplement: Table S2 — Set of mutated proteins by alanine scanning. Information of the mutated proteins and its catalytic residues, these are the elements of the positive group in test set B of xCMASA. (DOCX) [file pone.0108513.s002.docx]

Table S2**. Set of mutated proteins by alanine scanning.** Information of the mutated proteins and its catalytic residues, these are the elements of the positive group in test set B of xCMASA.

| **Protein name** | **Family name** | **Active sites** | **Mutated residue by ALA** |
| --- | --- | --- | --- |
| 1A8IA | 1A8IA | K568-R569-K574-T676 | 574 |
| 1AHPA | 1A8IA | K533-R534-K539-T641 | 534 |
| 1EM6A | 1A8IA | K568-R569-K574-T676 | 676 |
| 1GPAA | 1A8IA | K568-R569-K574-T676 | 574 |
| 1ACOA | 1ACOA | H101-H147-D165-H167-E262 | 101 |
| 1FGHA | 1ACOA | H101-H147-D165-H167-E262 | 101 |
| 1AFWA | 1AFWA | C125-H375-C403-G405 | 375 |
| 2C7YA | 1AFWA | C138-H393-C425-G427 | 427 |
| 2IIKA | 1AFWA | C123-H378-C409-G411 | 409 |
| 1A59A | 1AL6A | S191-H221-H269-D320 | 320 |
| 1AL6A | 1AL6A | S244-H274-H320-D375 | 375 |
| 1K3PA | 1AL6A | S234-H264-H305-D362 | 234 |
| 1O7XA | 1AL6A | S188-H218-H258-D313 | 218 |
| 2H12A | 1AL6A | S242-H272-H313-D371 | 272 |
| 2IFCA | 1AL6A | S192-H222-H262-D317 | 222 |
| 1AT1A | 1AT1A | R54-T55-R105-H134 | 54 |
| 1ML4A | 1AT1A | R57-T58-R107-H135 | 58 |
| 2BE7A | 1AT1A | R55-T56-R106-H134 | 55 |
| 1AW5A | 1AW5A | D131-S179-K210-K263 | 179 |
| 1B4EA | 1AW5A | D118-S165-K195-K247 | 165 |
| 1B4KA | 1AW5A | D127-S175-K205-K260 | 205 |
| 1E51A | 1AW5A | D120-S168-K199-K252 | 252 |
| 1H7OA | 1AW5A | D131-S179-K210-K263 | 210 |
| 1W1ZA | 1AW5A | D123-S170-K200-K253 | 123 |
| 1B8PA | 1BDMA | D162-R165-N189-H190 | 165 |
| 1BDMA | 1BDMA | D158-R161-N185-H186 | 161 |
| 1AQ7A | 1BITA | H57-D102-G193-S195-G196 | 193 |
| 1BITA | 1BITA | H57-D102-G193-S195-G196 | 196 |
| 1FN8A | 1BITA | H57-D102-G193-S195-G196 | 102 |
| 1FXYA | 1BITA | H57-D102-G193-S195-G196 | 193 |
| 2EEKA | 1BITA | H57-D102-G193-S195-G196 | 193 |
| 2EHHA | 1BITA | T43-T44-Y132-R137-K161 | 137 |
| 1BLSA | 1BLSA | S64-K67-Y150-E272-K315 | 272 |
| 1C3BA | 1BLSA | S64-K67-Y150-E272-K315 | 67 |
| 1BMFD | 1BMFD | K162-E188-R189-R356 | 162 |
| 1OHHD | 1BMFD | K162-E188-R189-R356 | 162 |
| 1SKYE | 1BMFD | K164-E190-R191-R352 | 191 |
| 2DPYA | 1BMFD | K188-E211-R212-R374 | 188 |
| 1BX4A | 1BX4A | R132-G297-A298-G299-D300 | 132 |
| 1DGMA | 1BX4A | R136-G315-A316-G317-D318 | 317 |
| 1LIJA | 1BX4A | R136-G315-A316-G317-D318 | 317 |
| 1LIOA | 1BX4A | R136-G315-A316-G317-D318 | 318 |
| 1BZCA | 1BZCA | D181-C215-R221-S222 | 215 |
| 1L8KA | 1BZCA | D182-C216-R222-S223 | 222 |
| 1WCHA | 1BZCA | D2378-C2408-R2414-S2415 | 2378 |
| 2QEPA | 1BZCA | D913-C945-R951-S952 | 952 |
| 1AH0A | 1C9WA | D43-Y48-K77-H110 | 43 |
| 1C9WA | 1C9WA | D43-Y48-K77-H110 | 48 |
| 2BGQA | 1C9WA | D55-Y60-K88-H121 | 88 |
| 1CGTA | 1CGTA | H140-R227-D229-E257-H327 | 229 |
| 1CKIA | 1CKIA | D131-K133-D135-N136-T181 | 133 |
| 1CSNA | 1CKIA | D131-K133-D135-N136-T181 | 133 |
| 1F3MC | 1CKIA | D389-K391-D393-N394-T427 | 391 |
| 2F2UA | 1CKIA | D214-K216-D218-N219-T253 | 218 |
| 1A0CA | 1CLKA | H100-E231-K233-H270-D306 | 233 |
| 1A0DA | 1CLKA | H98-E229-K231-H268-D304 | 229 |
| 1A0EA | 1CLKA | H100-E231-K233-H270-D306 | 231 |
| 1CLKA | 1CLKA | H53-E180-K182-H219-D254 | 219 |
| 1CMVA | 1CMVA | H63-S132-H157-R165 | 157 |
| 1NKKA | 1CMVA | H63-S132-H157-R165 | 157 |
| 1O6EA | 1CMVA | H48-S116-H139-R147 | 139 |
| 1CMXA | 1CMXA | Q84-C90-H166-D181 | 84 |
| 1UCHA | 1CMXA | Q89-C95-H169-D184 | 95 |
| 2ETLA | 1CMXA | Q84-C90-H161-D176 | 176 |
| 1BN6A | 1CV2A | D117-W118-E141-H283 | 141 |
| 1CV2A | 1CV2A | D108-W109-E132-H272 | 109 |
| 2O2HA | 1CV2A | D109-W110-E133-H273 | 110 |
| 1CVIA | 1CVIA | R1011-H1012-R1079-H1257-D1258 | 1011 |
| 1BHEA | 1CZFA | D202-D223-D224-H251 | 251 |
| 1CZFA | 1CZFA | D180-D201-D202-H223 | 201 |
| 1IA5A | 1CZFA | D159-D180-D181-H202 | 180 |
| 1NHCA | 1CZFA | D186-D207-D208-H229 | 229 |
| 2IQ7A | 1CZFA | D178-D199-D200-H221 | 221 |
| 1D3GA | 1D3GA | F149-S215-T218-K255 | 255 |
| 1F76A | 1D3GA | F115-S175-T178-K217 | 175 |
| 1TV5A | 1D3GA | F278-S345-T348-K429 | 345 |
| 1U0EA | 1D3GA | K210-R272-E357-H388-K518 | 357 |
| 1A7AA | 1D6OA | H55-D131-K186-D190-H301 | 186 |
| 1DL2A | 1DL2A | E132-R136-D275-E435 | 136 |
| 1FMIA | 1DL2A | E330-R334-D463-E599 | 463 |
| 1KKTA | 1DL2A | E122-R126-D267-E409 | 122 |
| 1NXCA | 1DL2A | E282-R286-D415-E549 | 282 |
| 1DBTA | 1DV7A | K33-D60-K62-D65 | 60 |
| 1DQWA | 1DV7A | K59-D91-K93-D96 | 93 |
| 1DV7A | 1DV7A | K42-D70-K72-D75 | 70 |
| 1EIXA | 1DV7A | K44-D71-K73-D76 | 76 |
| 1VQTA | 1DV7A | K26-D52-K54-D57 | 57 |
| 2CZ5A | 1DV7A | K29-D57-K59-D62 | 59 |
| 2D2RA | 1DV7A | R17-R26-R180-R186 | 186 |
| 2F84A | 1DV7A | K102-D136-K138-D141 | 138 |
| 2F8QA | 1DV7A | E149-H230-E259-D261 | 261 |
| 2YYTA | 1DV7A | K34-D61-K63-D66 | 63 |
| 1DXLA | 1DXLA | C45-C50-T215-H449-E454 | 50 |
| 1DYSA | 1DYSA | Y86-R91-D92-D139-D316 | 92 |
| 1TMLA | 1DYSA | Y73-R78-D79-D117-D265 | 117 |
| 1A33A | 1DYWA | R66-F71-Q74-N113-L133 | 113 |
| 1DYWA | 1DYWA | R62-F67-Q70-N109-L129 | 70 |
| 1IHGA | 1DYWA | F80-Q83-F122-N133-142L | 122 |
| 1ISTA | 1DYWA | R53-F58-Q61-N100-L120 | 58 |
| 1MZWA | 1DYWA | R67-F72-Q75-N114-L134 | 134 |
| 1MZYA | 1DYWA | F95-G97-D129-H177-H287 | 177 |
| 1QNGA | 1DYWA | R62-F67-Q70-N109-L129 | 67 |
| 1XWNA | 1DYWA | R55-F60-Q63-N102-L122 | 60 |
| 2A2NA | 1DYWA | R535-F540-Q543-N582-L602 | 582 |
| 2BITX | 1DYWA | R55-F60-Q63-N102-L122 | 102 |
| 2BJIA | 1DYWA | D1047-E1070-T1095-D1220 | 1070 |
| 2ESLA | 1DYWA | R89-F94-Q97-N136-L156 | 94 |
| 2ESMA | 1DYWA | D198-K200-D202-N203-T237 | 200 |
| 2GW2A | 1DYWA | R67-F72-Q75-N114-L134 | 134 |
| 2PLUA | 1DYWA | R78-F83-Q86-N125-L145 | 86 |
| 1E7PA | 1E7PA | H257-E294-R301-H369-R404 | 257 |
| 1BQGA | 1EC7A | K211-K213-D319-H345-D372 | 211 |
| 1EC7A | 1EC7A | K205-K207-D313-H339-D366 | 366 |
| 1EC9A | 1EC7A | K205-K207-D313-H339-D366 | 366 |
| 1CY0A | 1ECLA | E9-D111-Y319-H365 | 319 |
| 1ECLA | 1ECLA | E9-D111-Y319-H365 | 111 |
| 2GAIA | 1ECLA | E12-D84-Y288-H334 | 12 |
| 1A3HA | 1EDGA | N138-E139-H200-Y202-E228 | 202 |
| 1CECA | 1EDGA | N139-E140-H198-Y200-E280 | 280 |
| 1ECEA | 1EDGA | N161-E162-H238-Y240-E282 | 161 |
| 1EDGA | 1EDGA | N169-E170-H254-Y256-E307 | 254 |
| 1A39A | 1EG1A | E197-D199-E202-H213 | 202 |
| 1OVWA | 1EG1A | E197-D199-E202-H213 | 199 |
| 1EH6A | 1EH6A | N137-C145-H146-E172 | 145 |
| 1SFEA | 1EH6A | N138-C146-H147-E173 | 138 |
| 1WRJA | 1EH6A | N112-C120-H121-E146 | 112 |
| 1WS0A | 1EH6A | G46-Q51-L91-E133 | 91 |
| 1XBAA | 1EH6A | D494-A496-R498-N499 | 498 |
| 2G7HA | 1EH6A | N120-C128-H129-E154 | 120 |
| 1F6DA | 1F6DD | D95-E117-E131-H213 | 117 |
| 1F6DD | 1F6DD | D95-E117-E131-H213 | 131 |
| 1V4VA | 1F6DD | D100-E122-E136-H206 | 122 |
| 1V8BA | 1F6DD | H54-D134-K230-D234-H345 | 234 |
| 1VC4A | 1F6DD | E51-K112-E160-N181-S215 | 160 |
| 3BEOA | 1F6DD | D100-E122-E136-H209 | 209 |
| 1F75A | 1F75A | R33-R42-R197-R203 | 42 |
| 1JP3A | 1F75A | R30-R39-R194-R200 | 194 |
| 2VG2A | 1F75A | R80-R89-R244-R250 | 244 |
| 1FCQA | 1FCQA | D111-E113-Y184-Y227-W301 | 113 |
| 2ATMA | 1FCQA | D107-E109-Y180-Y223-W296 | 109 |
| 2AYNA | 1FCQA | N108-C113-H434-D450 | 434 |
| 2PE4A | 1FCQA | D129-E131-Y202-Y247-W321 | 129 |
| 1FJMA | 1FJMA | R96-N124-H125-R221-H248 | 124 |
| 1IT6A | 1FJMA | R96-N124-H125-R221-H248 | 248 |
| 1S70A | 1FJMA | R96-N124-H125-R221-H248 | 124 |
| 1S95A | 1FJMA | R275-N303-H304-R400-H427 | 427 |
| 1FUGA | 1FUGA | H14-K165-R244-K245-D271 | 244 |
| 1O90A | 1FUGA | H30-K182-R265-K266-D292 | 266 |
| 2P02A | 1FUGA | H51-K203-R286-K287-D313 | 287 |
| 1FUOA | 1FUOA | T187-H188-S318-K324-E331 | 187 |
| 1VDKA | 1FUOA | T187-H188-S318-K324-E331 | 324 |
| 1YFMA | 1FUOA | T212-H213-S343-K349-E356 | 349 |
| 1FX0A | 1FX0A | K176-Q201-K202-R366 | 202 |
| 1SKYB | 1FX0A | K175-Q200-K201-R365 | 175 |
| 2R9VA | 1FX0A | K176-Q201-K202-R366 | 176 |
| 1EVUA | 1G0DA | C314-H373-D396-Y560 | 314 |
| 1G0DA | 1G0DA | C272-H332-D355-Y515 | 332 |
| 1KV3A | 1G0DA | C277-H335-D358-Y516 | 335 |
| 1L9MA | 1G0DA | C272-H330-D353-Y525 | 272 |
| 1LARA | 1G0DA | E1428-D1490-H1521-C1522-R1528 | 1522 |
| 1C3XA | 1G2OA | H105-E108-T245-N246 | 108 |
| 1G2OA | 1G2OA | H90-E93-T230-N231 | 231 |
| 1M73E | 1G2OA | H86-E89-T242-N243 | 89 |
| 1MGTA | 1G2OA | N133-C141-H142-E167 | 141 |
| 1TCUA | 1G2OA | H88-E91-T244-N245 | 245 |
| 1ULAA | 1G2OA | H86-E89-T242-N243 | 86 |
| 1UOZA | 1G2OA | Y162-R167-D168-D206-D353 | 168 |
| 2P4SA | 1G2OA | H171-E174-T327-N328 | 174 |
| 1GEHA | 1GEHA | K163-K165-K189-D191-H281 | 163 |
| 1GEHA | 1GEHA | K163-K165-K189-D191-H281 | 281 |
| 1GERA | 1GERA | C42-K50-E181-H439-E444 | 50 |
| 1GETB | 1GERA | C42-K50-E181-H439-E444 | 181 |
| 1GK8A | 1GERA | K175-K177-D203-H294-H327 | 203 |
| 1ONFA | 1GERA | C39-K47-E189-H484-E489 | 484 |
| 1BWVA | 1GK8A | K175-K177-D203-H294-H327 | 327 |
| 1RBLA | 1GK8A | K175-K177-D203-H294-H327 | 175 |
| 1B30A | 1GOKA | E132-H210-E238-D240 | 210 |
| 1CLXA | 1GOKA | E127-H215-E246-D248 | 127 |
| 1E0VA | 1GOKA | E128-H207-E236-D238 | 207 |
| 1GOKA | 1GOKA | E131-H209-E237-D239 | 237 |
| 1HIZA | 1GOKA | E160-H237-E266-D268 | 237 |
| 1NQ6A | 1GOKA | E127-H206-E235-D237 | 206 |
| 1TA3B | 1GOKA | E128-H206-E236-D238 | 128 |
| 1CELA | 1GPIA | E212-D214-E217-H228 | 228 |
| 1GPIA | 1GPIA | E207-D209-E212-H223 | 223 |
| 1Q9HA | 1GPIA | E209-D211-E214-H225 | 214 |
| 1HVXA | 1HVXA | H106-R232-D234-H330-D331 | 234 |
| 2DIEA | 1HVXA | H107-R234-D236-H332-D333 | 107 |
| 2DP3A | 1HVXA | N11-K13-H96-E170-G176 | 11 |
| 1G8FA | 1I2DA | R197-H201-H204-R290 | 290 |
| 1I2DA | 1I2DA | R199-H203-H206-R292 | 292 |
| 1JHDA | 1I2DA | R201-H205-H208-R296 | 208 |
| 1ITKA | 1ITKA | R92-H96-D125-N126 | 92 |
| 1MWVA | 1ITKA | R108-H112-D141-N142 | 142 |
| 1SJ2A | 1ITKA | R104-H108-D137-N138 | 138 |
| 1UB2A | 1ITKA | R90-H94-D123-N124 | 123 |
| 1IICA | 1IYKA | N169-F170-L171-L455 | 171 |
| 1IYKA | 1IYKA | N175-F176-L177-L451 | 451 |
| 2NMTA | 1IYKA | N169-F170-L171-L455 | 455 |
| 1B3NA | 1J3NA | C163-H303-K335-H340-F398 | 335 |
| 1DD8A | 1J3NA | C163-H298-K328-H333-F390 | 328 |
| 1E5MA | 1J3NA | C167-H307-K339-H344-F401 | 344 |
| 1J3NA | 1J3NA | C161-H301-K333-H338-F394 | 338 |
| 1OX0A | 1J3NA | C164-H303-K332-H337-F394 | 337 |
| 1P3JA | 1J3NA | K13-R127-D162-D163-R171 | 171 |
| 2GP6A | 1J3NA | C170-H311-K341-H346-F403 | 170 |
| 1A9UA | 1JNKA | D150-K152-S154-N155-T185 | 152 |
| 1JNKA | 1JNKA | D189-K191-S193-N194-T226 | 193 |
| 1D0CA | 1K2RA | C186-R189-W358-E363 | 363 |
| 1K2RA | 1K2RA | C415-R418-W587-E592 | 592 |
| 1NSIA | 1K2RA | C200-R203-W372-E377 | 377 |
| 3NOSA | 1K2RA | C184-R187-W356-E361 | 187 |
| 1K49A | 1K4LA | D41-C66-D99-H136-E174 | 174 |
| 1K4LA | 1K4LA | D41-C66-D99-H136-E174 | 99 |
| 1TKSA | 1K4LA | D34-C59-D92-H128-E166 | 34 |
| 1D2KA | 1KFWA | D167-D169-E171-Y239 | 171 |
| 1GUVA | 1KFWA | D136-D138-E140-Y212 | 140 |
| 1ITXA | 1KFWA | D200-D202-E204-Y279 | 279 |
| 1KFWA | 1KFWA | D188-D190-E192-Y271 | 190 |
| 1W9PA | 1KFWA | D173-D175-E177-Y245 | 173 |
| 1BS4A | 1LMEA | G45-Q50-L91-E133 | 50 |
| 1BSJA | 1LMEA | G45-Q50-L91-E133 | 45 |
| 1IX1A | 1LMEA | G46-Q51-L93-E135 | 46 |
| 1LM4A | 1LMEA | G60-Q65-L112-E155 | 155 |
| 1LM6A | 1LMEA | G72-Q77-L131-E174 | 72 |
| 1LMEA | 1LMEA | G44-Q49-L88-E130 | 44 |
| 1SV2A | 1LMEA | G48-Q53-L102-E144 | 53 |
| 1V3YA | 1LMEA | G44-Q49-L103-E146 | 49 |
| 1ZXZA | 1LMEA | G49-Q54-L112-E154 | 49 |
| 2OKLA | 1LMEA | G60-Q65-L111-E154 | 65 |
| 1AR1A | 1M56A | H276-H411-H413-R473-R474 | 276 |
| 1M56A | 1M56A | H284-H419-H421-R481-R482 | 421 |
| 1OCCA | 1M56A | H240-H376-H378-R438-R439 | 240 |
| 1AMKA | 1MO0A | N11-K13-H95-E167-G173 | 173 |
| 1AW1A | 1MO0A | N9-K11-H97-E169-G175 | 175 |
| 1BTMA | 1MO0A | N8-K10-H94-E166-G172 | 172 |
| 1CI1A | 1MO0A | N12-K14-H96-E168-G174 | 14 |
| 1HG3A | 1MO0A | N12-K14-H96-E144-G150 | 150 |
| 1HTIA | 1MO0A | N11-K13-H95-E165-G171 | 11 |
| 1I45A | 1MO0A | N10-K12-H95-E165-G171 | 10 |
| 1LYXA | 1MO0A | N10-K12-H95-E165-G171 | 12 |
| 1M6JA | 1MO0A | N11-K13-H102-E174-G180 | 11 |
| 1MO0A | 1MO0A | N10-K12-H94-E164-G170 | 164 |
| 1SPQA | 1MO0A | N11-K13-H95-E165-G173 | 95 |
| 1TMHA | 1MO0A | N11-K13-H97-E169-G175 | 97 |
| 1W0MA | 1MO0A | N9-K11-H93-E141-G147 | 93 |
| 1YYAA | 1MO0A | N9-K11-H94-E166-G172 | 166 |
| 2I9EA | 1MO0A | N10-K12-H94-E164-G170 | 164 |
| 2JGQA | 1MO0A | N8-K10-H90-E159-G165 | 90 |
| 2VEIA | 1MO0A | N11-K13-H95-E167-G173 | 11 |
| 1ALQA | 1N9BA | S70-K73-S130-E166 | 70 |
| 1AXBA | 1N9BA | S70-K73-S130-E166 | 73 |
| 1BLCA | 1N9BA | S70-K73-S130-E166 | 73 |
| 1BSGA | 1N9BA | S70-K73-S130-E166 | 130 |
| 1BTLA | 1N9BA | S70-K73-S130-E166 | 166 |
| 1BUEA | 1N9BA | S70-K73-S130-E166 | 70 |
| 1E25A | 1N9BA | S70-K73-S130-E166 | 166 |
| 1G68A | 1N9BA | S70-K73-S130-E166 | 130 |
| 1HZOA | 1N9BA | S70-K73-S130-E166 | 70 |
| 1I2SA | 1N9BA | S70-K73-S130-E166 | 166 |
| 1N9BA | 1N9BA | S70-K73-S130-E166 | 73 |
| 2CC1A | 1N9BA | S70-K73-S130-E166 | 166 |
| 2GDNA | 1N9BA | S70-K73-S130-E166 | 166 |
| 1NDBA | 1NDBA | Y107-P120-H343-S554 | 107 |
| 1NDIA | 1NDBA | Y107-P120-H343-S554 | 107 |
| 1T7NA | 1NDBA | Y107-P120-H343-S554 | 554 |
| 1AQ8A | 1NIDA | F64-G66-D98-H145-H255 | 98 |
| 1KCBA | 1NIDA | F64-G66-D98-H145-H255 | 145 |
| 1NIDA | 1NIDA | F64-G66-D98-H145-H255 | 66 |
| 1J36A | 1O86A | H337-E368-E395-H497-Y507 | 368 |
| 1O86A | 1O86A | H353-E384-E411-H513-Y523 | 523 |
| 1O8AA | 1O86A | H353-E384-E411-H513-Y523 | 523 |
| 1IYXA | 1OEPA | E163-E204-K339-H367-K390 | 204 |
| 1OEPA | 1OEPA | E165-E208-K343-H371-K394 | 394 |
| 1W6TA | 1OEPA | E164-E205-K343-H371-K394 | 394 |
| 1P5HA | 1P5HA | Q17-E140-D169-G260-G261 | 169 |
| 1PDYA | 1P5HA | E166-E209-K344-H372-K395 | 395 |
| 1PQYA | 1P5HA | Q19-E142-D171-G250-G251 | 142 |
| 1PEMA | 1PEMA | C178-N386-C388-E390-C415 | 178 |
| 1ZYZA | 1PEMA | C218-N426-C428-E430-C443 | 430 |
| 3R1RA | 1PEMA | C225-N437-C439-E441-C462 | 437 |
| 1MTOA | 1PFKA | G11-R72-T125-D127-R171 | 11 |
| 1PFKA | 1PFKA | G11-R72-T125-D127-R171 | 72 |
| 1PG5A | 1PFKA | R51-T52-R101-H129 | 101 |
| 2HIGA | 1PFKA | G107-R173-T227-D229-R274 | 274 |
| 1QHFA | 1QHFA | H8-R59-E86-H181 | 86 |
| 1T8PA | 1QHFA | H11-R62-E89-H188 | 89 |
| 1YFKA | 1QHFA | H11-R62-E89-H186 | 11 |
| 1CRKA | 1QK1A | R127-E227-R231-R287-R315 | 315 |
| 1G0WA | 1QK1A | R132-E232-R236-R292-R320 | 236 |
| 1I0EA | 1QK1A | R132-E232-R236-R292-R320 | 232 |
| 1QK1A | 1QK1A | R127-E227-R231-R287-R315 | 127 |
| 1VRPA | 1QK1A | R132-E232-R236-R292-R320 | 292 |
| 2GL6A | 1QK1A | R166-E266-R270-R326-R354 | 266 |
| 1QAPA | 1QPNA | R118-K153-E214-D235 | 118 |
| 1QPNA | 1QPNA | R105-K140-E201-D222 | 222 |
| 1QPRA | 1QPNA | R105-K140-E201-D222 | 105 |
| 2B7NA | 1QPNA | R91-K126-E188-D209 | 188 |
| 2B9FA | 1QPNA | D137-K139-S141-N142-T185 | 139 |
| 2JBMA | 1QPNA | R104-K141-E203-D224 | 104 |
| 2JFNA | 1QPNA | D28-S29-C92-C204 | 28 |
| 1QWOA | 1QWOA | R62-R142-H338-D339 | 142 |
| 1BU8A | 1RP1A | F77-L153-D176-H263 | 77 |
| 1ETHA | 1RP1A | F78-L154-D177-H264 | 78 |
| 1GPLA | 1RP1A | F77-L153-D176-H263 | 77 |
| 1HPLA | 1RP1A | F77-L153-D176-H263 | 176 |
| 1LPAB | 1RP1A | F77-L153-D176-H263 | 77 |
| 1LQYA | 1RP1A | G60-Q65-L111-E154 | 65 |
| 1RP1A | 1RP1A | F77-L153-D176-H263 | 263 |
| 2OXEA | 1RP1A | F96-L172-D195-H282 | 96 |
| 2PPLA | 1RP1A | F96-L172-D194-H281 | 96 |
| 1RPNA | 1T2AA | T126-E128-Y150-K154 | 128 |
| 1T2AA | 1T2AA | T155-E157-Y179-K183 | 179 |
| 1T4CA | 1T2AA | Q17-E140-D169-G260-G261 | 17 |
| 1B3RA | 1V8BA | H54-D130-K185-D189-H300 | 185 |
| 1A53A | 1VC4A | E51-K110-E159-N180-S211 | 110 |
| 1DVEA | 1WE1A | H25-T135-D140-G143-G144 | 140 |
| 1N3UA | 1WE1A | H25-T135-D140-G143-G144 | 135 |
| 1WE1A | 1WE1A | H17-T126-D131-G134-G135 | 134 |
| 1WOVA | 1WE1A | H16-T125-D130-G133-G134 | 133 |
| 2Q32A | 1WE1A | H45-T155-D160-G163-G164 | 164 |
| 1B93A | 1WO8A | H19-G66-D91-H98-D101 | 91 |
| 1VMDA | 1WO8A | H23-G70-D95-H102-D105 | 70 |
| 1WO8A | 1WO8A | H9-G56-D81-H88-D91 | 88 |
| 1GQ8A | 1XG2A | Q113-Q135-D136-D157 | 157 |
| 1QJVA | 1XG2A | Q153-Q177-D178-D199 | 178 |
| 1XG2A | 1XG2A | Q109-Q131-D132-D153 | 109 |
| 1XQZA | 1XG2A | D167-K169-E171-N172-T204 | 172 |
| 1KWPA | 1Y8GA | D186-K188-E190-N191 | 188 |
| 1Y8GA | 1Y8GA | D175-K177-E179-N180 | 177 |
| 2H34A | 1Y8GA | D139-K141-E143-N144 | 143 |
| 2H6DA | 1Y8GA | D139-K141-E143-N144 | 143 |
| 2HW6A | 1Y8GA | D170-K172-E174-N175 | 172 |
| 1C3CA | 1YISA | T140-H141-K268-E275 | 268 |
| 1YISA | 1YISA | T154-H155-K291-E298 | 298 |
| 1YRPA | 1YISA | D139-K141-E143-N144-T180 | 143 |
| 1YVJA | 1YISA | D949-A951-R953-N954 | 951 |
| 2J91A | 1YISA | T158-H159-K295-E302 | 302 |
| 1GZKA | 1YRPA | D275-K277-E279-N280-T313 | 279 |
| 1H1WA | 1YRPA | D205-K207-E209-N210-T245 | 205 |
| 1HOWA | 1YRPA | D294-K296-E298-N299-T567 | 567 |
| 1IA8A | 1YRPA | D130-K132-E134-N135-T170 | 135 |
| 1IG1A | 1YRPA | D139-K141-E143-N144-T180 | 139 |
| 1VZOA | 1YRPA | D177-K179-E181-N182-T216 | 181 |
| 1W0IA | 1YRPA | C209-H350-K384-H389-F447 | 384 |
| 1WAKA | 1YRPA | D213-K215-E217-N218-T514 | 215 |
| 1WMKA | 1YRPA | D139-K141-E143-N144-T180 | 144 |
| 2BFXA | 1YRPA | D216-K218-E220-N221-T252 | 216 |
| 2C47A | 1YRPA | D125-K127-E129-N130-T175 | 130 |
| 2C4MA | 1YRPA | K522-R523-K528-T630 | 522 |
| 2CHLA | 1YRPA | D151-K153-E155-N156-T201 | 201 |
| 2CMWA | 1YRPA | D164-K166-E168-N169-T214 | 166 |
| 2CN5A | 1YRPA | D347-K349-E351-N352-T387 | 351 |
| 2CWXA | 1YRPA | K160-K162-K186-D188-H278 | 160 |
| 2IWIA | 1YRPA | D163-K165-E167-N168-T200 | 163 |
| 2PZIA | 1YRPA | D276-K278-E280-N281-T309 | 278 |
| 1CM0A | 1Z4RA | E570-I571-V572-I637-Y640 | 570 |
| 1Z4RA | 1Z4RA | E575-I576-V577-I642-Y645 | 576 |
| 1AKEA | 1ZIOA | K13-R156-D158-D159-R167 | 159 |
| 1P4SA | 1ZIOA | K13-R127-D131-D132-R140 | 132 |
| 1S3GA | 1ZIOA | K13-R160-D162-D163-R171 | 163 |
| 1ZIOA | 1ZIOA | K13-R127-D162-D163-R171 | 163 |
| 1ZKJA | 1ZIOA | S65-K68-Y151-E272-K312 | 151 |
| 2RGXA | 1ZIOA | K13-R150-D152-D153-R161 | 150 |
| 1B73A | 1ZUWA | D7-S8-C70-C178 | 8 |
| 1ZUWA | 1ZUWA | D10-S11-C74-C185 | 10 |
| 1ZXXA | 1ZUWA | G11-R72-T125-D127-R171 | 171 |
| 2JFOA | 1ZUWA | D11-S12-C74-C185 | 12 |
| 2JFQA | 1ZUWA | D9-S10-C72-C184 | 9 |
| 2JFUA | 1ZUWA | D14-S15-C77-C188 | 188 |
| 2JFXA | 1ZUWA | D7-S8-C70-C181 | 181 |
| 2OHGA | 1ZUWA | D10-S11-C73-C183 | 183 |
| 1FZTA | 2A6PA | H15-R66-E93-H163 | 15 |
| 1RIIA | 2A6PA | H12-R63-E90-H183 | 90 |
| 2A6PA | 2A6PA | H13-R64-E85-H147 | 64 |
| 1CI7A | 2AAZA | C173-S200-D202-N210-H240 | 210 |
| 2AAZA | 2AAZA | C187-S217-D219-N227-H257 | 257 |
| 1BYGA | 2B7AA | D314-A316-R318-N319 | 318 |
| 1K2PA | 2B7AA | D521-A523-R525-N526 | 521 |
| 1MP8A | 2B7AA | D546-A548-R550-N551 | 546 |
| 1SM2A | 2B7AA | D482-A484-R486-N487 | 487 |
| 1U46A | 2B7AA | D252-A254-R256-N257 | 257 |
| 2B7AA | 2B7AA | D976-A978-R980-N981 | 978 |
| 3BKBA | 2B7AA | D683-A685-R687-N688 | 683 |
| 1EDOA | 2C07A | N126-S154-Q164-Y167-K171 | 126 |
| 1EG1A | 2C07A | E196-D198-E201-H212 | 198 |
| 2C07A | 2C07A | N171-S199-Q209-Y212-K216 | 171 |
| 2P68A | 2C07A | N116-S144-Q154-Y157-K161 | 116 |
| 1AD5A | 2DQ7X | D386-R388-A390-N391 | 391 |
| 1FMKA | 2DQ7X | D386-R388-A390-N391 | 388 |
| 1QPCA | 2DQ7X | D364-R366-A368-N369 | 366 |
| 2DQ7X | 2DQ7X | D130-R132-A134-N135 | 130 |
| 2E28A | 2DQ7X | R33-K221-T279-S313-E315 | 33 |
| 1EBLA | 2EBDA | C112-F157-H244-N274 | 112 |
| 1HZPA | 2EBDA | C112-F157-H244-N274 | 157 |
| 1MZJA | 2EBDA | C121-F166-H257-N288 | 121 |
| 1ZOWA | 2EBDA | C112-F157-H238-N268 | 268 |
| 2EBDA | 2EBDA | C111-F156-H236-N266 | 236 |
| 1XXXA | 2EHHA | T54-T55-Y143-R148-K171 | 148 |
| 1XYZA | 2EHHA | E645-H723-E754-D756 | 756 |
| 2RFGA | 2EHHA | T43-T44-Y132-R137-K160 | 137 |
| 2YXGA | 2EHHA | T43-T44-Y132-R137-K161 | 132 |
| 2BVAA | 2F57A | D440-K442-D444-S445-T478 | 444 |
| 2C30A | 2F57A | D526-K528-D530-S531-T564 | 531 |
| 2F57A | 2F57A | D568-K570-D572-S573-T606 | 570 |
| 1U2PA | 2FEKA | C11-C16-R17-D126 | 11 |
| 1ZGGA | 2FEKA | C7-C12-R13-D118 | 118 |
| 1ZINA | 2FEKA | K13-R127-D162-D163-R171 | 163 |
| 2FEKA | 2FEKA | C9-C14-R15-D115 | 115 |
| 1NB8A | 2GFOA | N218-C223-H464-D481 | 464 |
| 1NBFA | 2GFOA | N218-C223-H464-D481 | 218 |
| 2GFOA | 2GFOA | N781-C786-H1067-D1084 | 1067 |
| 2GSAA | 2GSAA | Y150-F157-D245-K273 | 245 |
| 1H2BA | 2HCYA | H55-T56-H59-W65 | 55 |
| 1RJWA | 2HCYA | H39-T40-H43-W49 | 43 |
| 2HCYA | 2HCYA | H44-T45-H48-W54 | 44 |
| 1A1SA | 2I6UA | R58-T59-R107-H134-Q137 | 107 |
| 1C9YA | 2I6UA | R92-T93-R141-H168-Q171 | 168 |
| 1DXHA | 2I6UA | R58-T59-R107-H134-Q137 | 107 |
| 1VLVA | 2I6UA | R59-T60-R108-H135-Q138 | 60 |
| 2I6UA | 2I6UA | R52-T53-R101-H128-Q131 | 128 |
| 2FSSA | 2NACA | N119-R258-Q287-H311 | 119 |
| 2GO1A | 2NACA | N146-R284-Q313-H332 | 146 |
| 2GSDA | 2NACA | N146-R284-Q313-H332 | 146 |
| 2NACA | 2NACA | N146-R284-Q313-H332 | 332 |
| 1A8PA | 2OK7A | Y53-S54-C219-E252 | 53 |
| 1QFYA | 2OK7A | Y89-S90-C266-E306 | 90 |
| 2B5OA | 2OK7A | Y181-S182-C360-E400 | 182 |
| 2OK7A | 2OK7A | Y103-S104-C284-E314 | 103 |
| 1E9IA | 2PA6A | E167-E208-K341-H369-K392 | 392 |
| 1EBGA | 2PA6A | E168-E211-K345-H373-K396 | 345 |
| 2PA6A | 2PA6A | E170-E213-K339-H367-K390 | 213 |
| 1PGJA | 2PGDA | G132-K185-N189-E192 | 132 |
| 1PGNA | 2PGDA | G130-K183-N187-E190 | 130 |
| 2IYOA | 2PGDA | G130-K184-N188-E191 | 130 |
| 2P4QA | 2PGDA | G130-K182-N186-E189 | 186 |
| 2PGDA | 2PGDA | G130-K183-N187-E190 | 190 |
| 1DLIA | 2Q3EA | T118-E145-K204-N208-D264 | 118 |
| 2O3JA | 2Q3EA | T136-E172-K227-N231-D287 | 136 |
| 2Q3EA | 2Q3EA | T131-E165-K220-N224-D280 | 165 |
| 1B0ZA | 2Q8NA | K139-R202-E285-H306-K420 | 420 |
| 1DQRA | 2Q8NA | K210-R272-E357-H388-K518 | 357 |
| 1Q50A | 2Q8NA | K258-R325-E410-H441-K569 | 441 |
| 1ZZGA | 2Q8NA | K125-R187-E267-H293-K406 | 293 |
| 2O2CA | 2Q8NA | K259-R326-E411-H442-K571 | 442 |
| 2Q8NA | 2Q8NA | K137-R198-E281-H310-K422 | 198 |
| 2Q74A | 2QFLA | D60-E83-T109-D235 | 83 |
| 2QFLA | 2QFLA | D44-E67-T89-D212 | 89 |
| 1GS5A | 2RD5A | K8-G11-G45-K217 | 217 |
| 1OH9A | 2RD5A | K8-G11-G45-K217 | 217 |
| 2AP9A | 2RD5A | K36-G39-G72-K253 | 39 |
| 2BTYA | 2RD5A | K27-G30-G63-K237 | 237 |
| 2BUFA | 2RD5A | K33-G36-G69-K255 | 255 |
| 2RD5A | 2RD5A | K41-G44-G77-K255 | 77 |
| 1FVRA | 2REIA | D964-A966-R968-N969 | 968 |
| 1GAGA | 2REIA | D1132-A1134-R1136-N1137 | 1132 |
| 1IR3A | 2REIA | D1132-A1134-R1136-N1137 | 1137 |
| 1JPAA | 2REIA | D754-A756-R758-N759 | 754 |
| 1JQHA | 2REIA | D1135-A1137-R1139-N1140 | 1139 |
| 1LUFA | 2REIA | D724-A726-R728-N729 | 726 |
| 1M14A | 2REIA | D813-A815-R817-N818 | 815 |
| 1MQBA | 2REIA | D739-A741-R743-N744 | 739 |
| 1PKGA | 2REIA | D792-A794-R796-N797 | 792 |
| 1R0PA | 2REIA | D1204-A1206-R1208-N1209 | 1204 |
| 1RJBA | 2REIA | D811-A813-R815-N816 | 813 |
| 1VR2A | 2REIA | D1028-A1030-R1032-N1033 | 1033 |
| 1Y6AA | 2REIA | D1026-A1028-R1030-N1031 | 1026 |
| 2GSFA | 2REIA | D746-A748-R750-N751 | 746 |
| 2HELA | 2REIA | D746-A748-R750-N751 | 751 |
| 2I0VA | 2REIA | D778-A780-R782-N783 | 778 |
| 2IVSA | 2REIA | D874-A876-R878-N879 | 878 |
| 2P0CA | 2REIA | D723-A725-R727-N728 | 728 |
| 2R2PA | 2REIA | D800-A802-R804-N805 | 802 |
| 2REIA | 2REIA | D758-A760-R762-N763 | 760 |
| 1A3WA | 2VGBA | R49-K240-T298-S332-E334 | 49 |
| 1A49A | 2VGBA | R72-K269-T327-S361-E363 | 361 |
| 1E0TA | 2VGBA | R32-K220-T278-S312-E314 | 32 |
| 1PKLA | 2VGBA | R49-K238-T296-S330-E332 | 332 |
| 1PKNA | 2VGBA | R72-K269-T327-S361-E363 | 269 |
| 2VGBA | 2VGBA | R116-K313-T371-S405-E407 | 313 |
